# Return of the Tbx5; lineage-tracing reveals ventricular cardiomyocyte-like precursors in the injured adult mammalian heart

**DOI:** 10.1038/s41536-023-00280-9

**Published:** 2023-03-03

**Authors:** Panagiota Siatra, Giannis Vatsellas, Athanasia Chatzianastasiou, Evangelos Balafas, Theodora Manolakou, Andreas Papapetropoulos, Anna Agapaki, Eleni-Taxiarchia Mouchtouri, Prashant J. Ruchaya, Artemis G. Korovesi, Manolis Mavroidis, Dimitrios Thanos, Dimitris Beis, Ioannis Kokkinopoulos

**Affiliations:** 1 Center for Clinical, Experimental Surgery and Translational Research, Biomedical Research Foundation, Academy of Athens, Athens, Greece; 2 Center for Basic Research, Biomedical Research Foundation, Academy of Athens, Athens, Greece; 3 Greek Genome Center, Biomedical Research Foundation, Academy of Athens, Athens, Greece; 4 Department of Pharmacy, Laboratory of Pharmacology, National and Kapodistrian University of Athens, Athens, Greece; 5 Histochemistry Facility, Biomedical Research Foundation, Academy of Athens, Athens, Greece; 6 School of Health, Sport and Biosciences, University of East London, London, UK

**Keywords:** Cell biology, Stem cells

## Abstract

The single curative measure for heart failure patients is a heart transplantation, which is limited due to a shortage of donors, the need for immunosuppression and economic costs. Therefore, there is an urgent unmet need for identifying cell populations capable of cardiac regeneration that we will be able to trace and monitor. Injury to the adult mammalian cardiac muscle, often leads to a heart attack through the irreversible loss of a large number of cardiomyocytes, due to an idle regenerative capability. Recent reports in zebrafish indicate that Tbx5a is a vital transcription factor for cardiomyocyte regeneration. Preclinical data underscore the cardioprotective role of Tbx5 upon heart failure. Data from our earlier murine developmental studies have identified a prominent unipotent Tbx5-expressing embryonic cardiac precursor cell population able to form cardiomyocytes, in vivo, in vitro and ex vivo. Using a developmental approach to an adult heart injury model and by employing a lineage-tracing mouse model as well as the use of single-cell RNA-seq technology, we identify a Tbx5-expressing ventricular cardiomyocyte-like precursor population, in the injured adult mammalian heart. The transcriptional profile of that precursor cell population is closer to that of neonatal than embryonic cardiomyocyte precursors. Tbx5, a cardinal cardiac development transcription factor, lies in the center of a ventricular adult precursor cell population, which seems to be affected by neurohormonal spatiotemporal cues. The identification of a Tbx5-specific cardiomyocyte precursor-like cell population, which is capable of dedifferentiating and potentially deploying a cardiomyocyte regenerative program, provides a clear target cell population for translationally-relevant heart interventional studies.

## Introduction

According to the World Health Organization, heart failure is the major cause of death in industrialized countries with an estimated 17.3 million deaths per year due to cardiovascular disease, representing 30% of all global deaths^1^. Human heart regeneration is one of the most critical unmet clinical needs at a global level. Congenital heart defects (CHDs) are usually apparent at birth, characterized by structural abnormalities, such as atrial or ventricular septation defects, electrical conduction abnormalities or cardiomyopathies. One of the primary causes of cardiomyopathies is the loss and/or damage of heart muscle cells, termed cardiomyocytes (CM). In order to replenish the lost/damaged cells, an appropriate source is needed as a cell-replacement therapeutic approach. An attractive candidate is cardiac precursor cells (CPCs), which could be driven to give rise to mature CMs.

During development, the CM lineage is highly specialized, comprising cardiac progenitors allocated in a discrete and temporal order^2^. At embryonic day (E) 7.5 in mice^3,4^, the heart tube is the initial structure that eventually gives rise to the heart proper. It is populated by two distinct sets of cardiac progenitors derived from two anatomical regions; the first heart field (FHF also known as the cardiac crescent) which will give rise to the left ventricle (LV) and parts of the atria, and the second heart field (SHF) that contributes towards the right ventricle, outflow tract and the remaining parts of the atria, including the septum^3–6^. These fields are genetically distinguishable, at E7.5, by expression of specific transcription factors (TF)^3,6,7^. After birth, most CMs are acytokinetic and have undergone terminal differentiation. However, recent studies have shown that the adult heart exhibits a capacity, albeit limited, to generate new CMs^8^. Carbon-14 birth-dating studies have suggested that around 40% of CMs are replaced over an entire life span, while IdU-labeling raises this percentage to 100%, in humans^9^, with a 1% per annum of CM turnover in the mammalian heart^10–13^. Tbx5, the T-box TF haploinsufficient in Holt-Oram syndrome, is one of the cardinal TFs essential for cardiac development and adult CM formation both in vivo and in vitro^14–18^.

Cardiomyocyte renewal in mammals could potentially be enabled *via* CM dedifferentiation and subsequent proliferation, as in the case of urodele amphibians and zebrafish^19^ Recent experiments performed in the adult zebrafish, reported that re-expression of *tbx5a* was essential for complete heart regeneration, upon ventricular ablation^20–22^. Thus, the Tbx5 transcriptional network is crucial not only for initiating early cardiac specification but to also prime the cardiac regenerative program, at least in adult lower vertebrates (ref. ^14^ and references within). While the priming of a resident CM proliferation program is now a leading therapeutic goal, the importance of Tbx5 in adult and postnatal mammalian heart ventricle regeneration has not been examined.

By employing a BAC *Tbx5*^*CreERT2/CreERT2*^ transgene injury heart model^23^, we report the presence of cardiac cells that have over-activated Tbx5 following myocardial injury, with markers shown to be expressed in early cardiovascular precursors. Tbx5 transcription is controlled by a positive feedback loop in early murine heart development^23^. Therefore, Tbx5 overexpression upon injury could be one of the early attempts for priming regulatory networks important for CM dedifferentiation, division and/or differentiation in mice and humans^16–18^.

## Results

### In vitro mESC-derived CPC follow a similar cardiac developmental program to in vivo early embryo CPCs

**Fig. 1:**
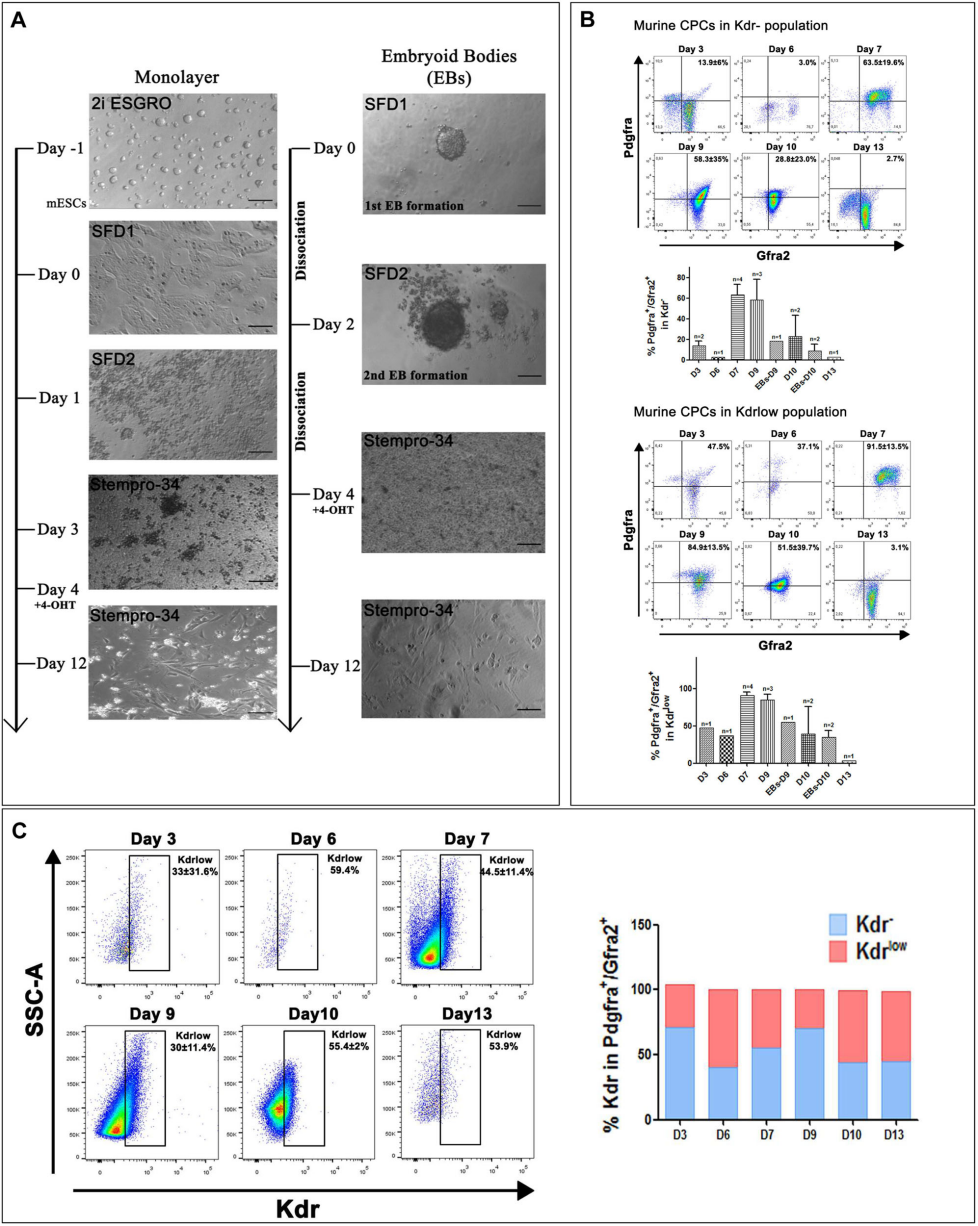
Enrichment and optimization of in vitro mESC-derived CPC enrichment. **A** Representative microphotographs depicting stages of differentiation of murine ground state *Tbx5*^*Cre*^*;R26R*^*eYFP/eYFP*^ mESC cultured under specified cardiomyocyte differentiation conditions, either as monolayers or as embryoid bodies. **B** Flow cytometric analysis using three surface markers Pdgfra, Kdr and Gfra2 on different days of cardiomyocyte differentiation indicates an CPC enrichment window between days 7 and 9. **C** The expression of Kdr is dynamic and defines two waves of CPC in the current differentiation protocol. *N* = 1–4. Error bars = SEM.

**Fig. 2:**
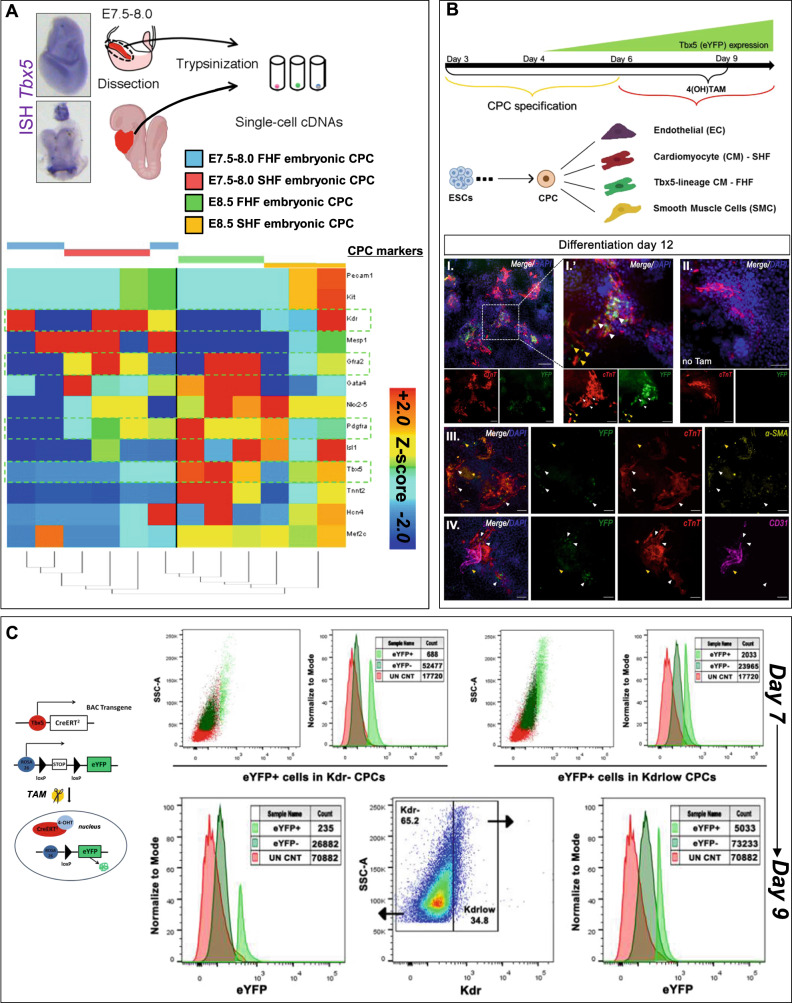
Tbx5 expression is enriched in CPC-derived cardiomyocytes. **A** Tbx5 expression in E7.5-E8.0 and E8.5 whole embryos according to mRNA in situ hybridization. Single-cell RNA-seq analysis of cardiac cells from E7.5-E8.0 and E8.5 embryos for known cardiac progenitor cell genes. **B** Representative microphotographs of 4OH-TAM-induced YFP (Tbx5-lineage tracing) expression (α-GFP, green) on cardiomyocytes (cTnT, red), endothelial cells (CD31, purple) and smooth muscle cells (α-SMA, yellow) that have differentiated from mESC-derived CPC on day 12. *N* = 5. **C** Flow cytometric analysis indicates that BAC *Tbx5*^*Cre*^*;R26R*^*eYFP/eYFP*^ insert can lineage trace Tbx5-expressing cells within the CPC population. *N* = 5. Scale bar = 10 μm.

We set to establish an in vitro mesodermal/cardiac differentiation pipeline that would pinpoint an optimal developmental window where CPCs could be examined and collected for further studies (Fig. 1). Based on a previously established CPCs differentiation system^24^, we were able to enrich for FHF and SHF CPC populations based on their surface expression profile. Murine BAC *Tbx5*^*CreERT2*^*/Rosa26R*^*eYFP/eYFP*^ ESCs were subjected to cardiac differentiation, while 4-hydroxytamoxifen (4OH-TΑΜ) was added from day 4 onwards (Fig. 1A). When CPCs were allowed to differentiate for up to 12 days in vitro, under non-FBS defined culture conditions, YFP (Tbx5-lineage traced) expression was observed in cTnT^+^ cells but never into endothelial nor smooth muscle cells (Fig. 2B). In suboptimal differentiation conditions, there was an absence of YFP^+^ with a subsequent decrease in cTnT^+^ cells, in total (Supplementary Fig. 1B).

Flow cytometric analysis indicated that Pdgfra^+^/Gfra2^+^/Kdr^low/+^ CPCs were enriched between days 7 and 9 (Fig. 1B and Supplementary Fig. 1A). Interestingly, in our in vitro culture system, Kdr surface marker pinpointed to two potential “waves” of Pdgfra^+^/Gfra2^+^ CPCs (Fig. 1C). To address this finding, we revisited previously published data from our lab concerning single-cell RNA-seq obtained from in vivo cardiac CPCs on different early embryonic stages^23^ (Fig. 2A). Gene expression meta-analysis indicated that embryonic day 8.5 (E8.5) CPC showed a decreased *Kdr* expression, when compared to E7.5 CPCs, in an inversely proportional manner to *Tbx5* expression, in vivo (Fig. 2A). The expression of YFP after 4OH-TAM administration, indicated that both Kdr^low/+^ and Kdr^**−**^ CPC (Pdgfra^+^/Gfra2^+^) subpopulations possessed YFP^+^ CPCs, yet only the Kdr^low/+^/Gfra2^+^/Pdgfra^+^/YFP^+^ CPC population increased from days 7 to 9 (Fig. 2C).

These data indicate that the in vitro mESC-derived CPCs’ surface marker expression profile follows a gene expression profile similar to that of in vivo CPCs involved in early cardiac embryonic development, with Tbx5^+^ CPCs to promote a unipotent CM-like fate both in vivo and in vitro.

### Reactivation of the TF Tbx5 in the adult injured mammalian heart

**Fig. 3:**
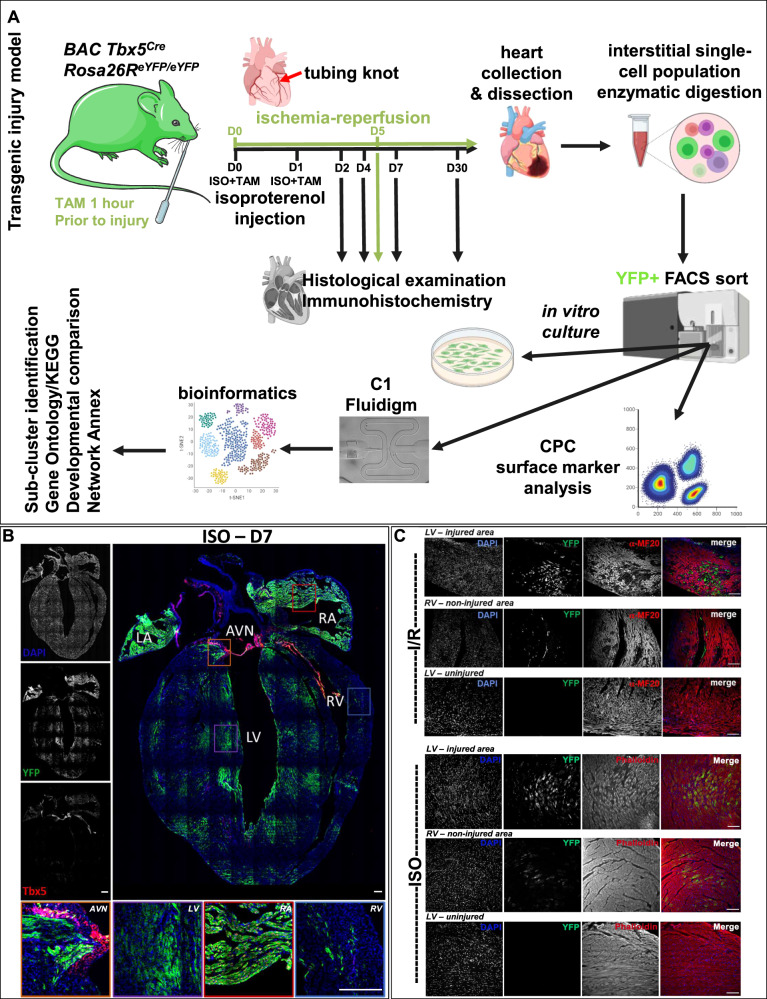
Tbx5 re-activation in the adult murine heart. **A** Schematic representation of the experimental pipeline for Tbx5-lineage (YFP^+^) tracing cell analysis in the adult injured murine heart. **B** A collage of an ISO-injured adult heart on D7 after injury. Key- LV Left Ventricle, RV Right Ventricle, RA Right Atrium, LA Left Atrium, AVN Atrioventricular Node, SAN Sinoatrial Node. Higher magnification inserts indicating the localization of YFP^+^ (α-GFP) cells in sites of ventricular injury, while YFP^+^Tbx5^+^ and Tbx5^+^YFP^−^ where only located in the atria and the nodes. **C** Representative microphotographs of adult hearts 5 days after I/R and 7 days after ISO injury, indicating YFP^+^ cells (α-GFP, green) and cardiomyocytes [MF20 or phalloidin/F-actin, red]. *N* = 2–3 hearts per condition and *n* > 6 sections per heart examined. Scale bar = 10 μm.

**Fig. 4:**
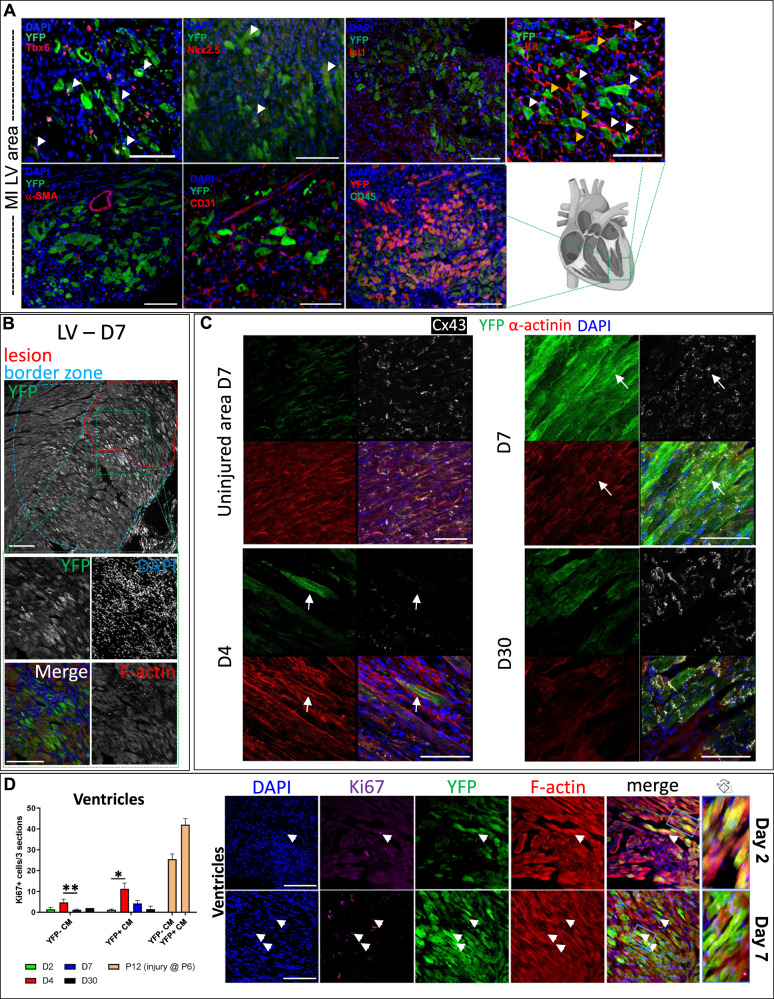
In situ characterization of YFP^+^ cells upon heart injury. **A** Representative microphotographs of immunohistochemistry performed in adult injured heart cryosections on day 5 after I/R. **B** Representative microphotographs of a lesion site where increased YFP^+^ (α-GFP, greyscale) CMs are present in the lesion and border zones. Higher magnification inserts indicate YFP^+^F-actin^+^ cardiac cells with a CM morphology located in and around the lesion site. **C** Representative microphotographs of lesions in adult injured hearts 4, 7 (and injured area), as well as 30 days post injury; YFP^+^-expressing cells (α-GFP, green), cardiomyocyte sarcomeres (α-actinin, red), gap-junction protein Connexin-43 (Cx43, white) and nuclear dye (DAPI, blue). Scale bars = as indicated. **D** XY graph depicting Ki67^+^ cells per section in YFP^+^ (α-GFP) and YFP^−^ CM in adult injured hearts, 2, 4 and 7 days post-injury, as well as 7 days post-injury in P6 pups. YFP^+^-expressing cells (α-GFP, green), cardiomyocytes (F-actin/Phalloidin, red), cycling cells (Κi67, magenta) and nuclear dye (DAPI, blue). Arrowheads indicative of co-localization of YFP^+^-expressing cells (α-GFP, green), cardiomyocytes (F-actin/Phalloidin, red), cycling cells (Κi67, magenta) and nuclear dye (DAPI, blue). Blue insert magnifies on a representative YFP^+^ (α-GFP) cell that has altered sarcomere striation designated by F-actin, when compared to an adjacent YFP^−^F-actin^+^ CM. *D2 vs D4 *p* = 0.0019, **D4 vs D7 *p* = 0.009583. Student’s T-test. *N* = 1–3 hearts per time-point and *n* > 6 sections per heart examined. Scale bar = 10 μm. Error bars = SEM.

Using a BAC *Tbx5*^*CreERT2/CreERT2*^*/Rosa26R*^*eYFP/eYFP*^ transgene^23^, we employed two adult heart injury murine models; (i) ischemia/reperfusion (I/R) induced MI, and (ii) chemical induced MI using ISO intraperitoneal (i.p.) injections to promote cardiomyocyte lesions in the cardiac muscle^25^ (Fig. 3A). Cardiac injury was confirmed using Haematoxylin and Eosin as well as Masson’s trichrome staining (Supplementary Fig. 2A, B). Immunohistochemical analysis indicated that Tbx5^+^/YFP^+^ cells were present in the injured ventricles four days after injury, while Tbx5^−^YFP^+^ cells were present in injured ventricles on days 7 and 30 post-injury; no Tbx5^+^/YFP^+^ were detected in uninjured ventricles, as expected^26^ (Fig. 3B and Supplementary Figs. 3, 4). No eYFP^+^ cells were readily observed in uninjured adult LV (Fig. 3C). To characterize these YFP^+^ further, adult injured heart immunohistochemistry indicated their co-expression with cardiogenic precursor markers such as Tbx5, Nkx2–5, but not Isl1. In addition, YFP^+^ did not co-localize with classical smooth muscle cell (α-SMA) nor immune cell markers (CD45), while CD31 and C-kit protein expression was observed in both YFP^+^ and YFP^−^ cells (Fig. 4A). By investigating the whole heart after injury, the presence of YFP^+^ CM was primarily observed around designated lesion sites^27,28^ (Fig. 4B). Confocal imaging indicated a disorganization of those YFP^+^ cells’ sarcomere structure and gap junctions following injury, peaking around days 4–7, while on some YFP^+^ CM, gap junctions were apparent by day 30 (Fig. 4C).

Immunohistochemical analysis was indicative of CM nuclei positive for the cell cycle protein Ki67 nuclei, yet with no apparent correlation to sarcomere disorganization neither on D4 nor D7. It is noted that no CM mitotic/cytokinetic event was observed. Further analysis at different time points indicated that some YFP^+^ CMs showed a low, yet persistent expression of Ki67, when compared to YFP^−^ CMs (Fig. 4D). These results are in line with recent findings of Ki67 expression in adult CM populations^29^.

**Fig. 5:**
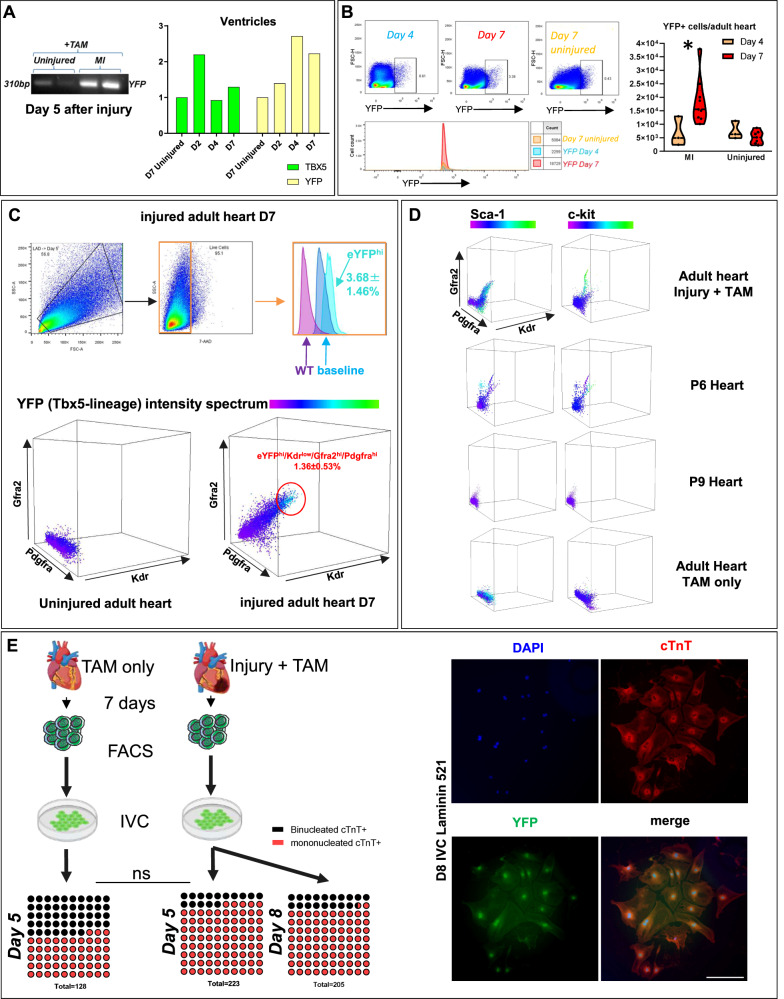
CPC surface analysis of YFP^+^ cells. **A** Real-time PCR analysis of *Yfp* and *Tbx5* transcripts in the ventricles of the adult heart in different time-points. **B** Flow cytometry acquisition of YFP^+^ cells from different time-points following cardiac injury. *N* = 3–6 hearts. **C**, **D** Representative two- and three-dimensional graphs from flow cytometric analysis of Pdgfra^+^Kdr^low/+^Gfra2^+^ adult, postnatal days 6 and 9 heart and their co-expression with YFP (Tbx5-tracing), Sca-1 and c-Kit. *N* = 9. Key-CP; cardiomyocyte precursor, CM; cardiomyocyte, EC; endothelial cell. **E** YFP^+^ cells were collected 7 days after chemical injury and cultured in CM differentiating conditions for 5 and 8 days. Only YFP^+^ from the injured heart were able to differentiate into CM-like cells. Measurement of YFP^+^/cTnT^+^ mononucleated and binucleated cells 5 and 8 days in vitro culture. *N* = 5–6 independent cultures. Mann–Whitney test, *p* = 0.0519 (ns = not statistically significant) for D5 binucleated/mononucleated ratio.

Flow cytometric analysis was performed in order to collect eYFP^+^ single cells from 2, 4, 5 and 7 days post-injury. The expression of YFP and Tbx5 transcripts in the atria and ventricles of the adult injured heart were also confirmed by qPCR (Fig. 5A, B and Supplementary Figs. 5A, 9). The eYFP protein expression was validated in cardiac ventricular cell populations in the injured hearts, with a peak YFP expression 7 days after injury (Fig. 5B).

The finding that an embryonic TF such as Tbx5 is being reactivated in the adult injured heart, led us to investigate whether the well-documented cell-surface embryonic CPC markers^30,31^, may be able to tag an adult cardiac cell subpopulation. FACS analysis was performed on cells collected from adult lungs and hearts (treated with Tamoxifen only) or injured adult hearts, as well as hearts derived from postnatal (P) days 6 and 9. Results showed that the YFP^+^ cells are a part of a wider Kdr^low/+^/Gfra2^+^/Pdgfra^+^ adult cardiac cell population (Fig. 5C and Supplementary Fig. 5B). Our data also showed that a Kdr^low/+^/Gfra2^+^/Pdgfra^+^ CPC subpopulation was detected only in cells derived from P6 whole heart tissue, but were near-absent in P9 hearts, neither in uninjured adult murine hearts nor lungs (Fig. 5D and Supplementary Fig. 5C). Of note, Sca-1 was detected, but could not be designated in Kdr^low/+^/Gfra2^+^/Pdgfra^+^/YFP^+/−^ cells only, while c-kit was not detected in our adult CPCs, yet present in P6 CPC (Kdr^low/+^/Gfra2^+^/Pdgfra^+^), as shown previously^32^.

Recent studies have demonstrated that CM polyploidy is relevant to their regenerative capacity, with polynucleation acting as a barrier against regeneration^33^. Collected YFP^+^ adult cells were cultured in vitro 7 days after injury, for 5 and 8 days (Fig. 5E). After 5 days in culture, an increased frequency of a mononucleated YFP^+^cTnT^+^ cell population in cells collected from the whole injured heart, in comparison to the whole uninjured heart, was observed (50.84 ± 17.9% SD uninjured vs 78.06 ± 13.9% SD, injured on Day 5).

Taken together, these data indicate that a Tbx5-expressing potentially precursor CM population is activated upon myocardial injury, in the adult mammalian heart.

### Injury-induced YFP+ CM precursors may support a reparative potential of the adult CM ventricular tissue

In order to assess the pathophysiology of Tbx5-expressing heart cells, we employed an mESC Tbx5-KO primary cell line, from which we attempted to obtain CPCs using a defined differentiation medium^23,24^. We observed a delay in the early Kdr^low/+^/Gfra2^+^/Pdgfra^+^ CPC formation (Day 7). This postponement was compensated later on (Day 10) (Supplementary Fig. 6A). To assess the potential pathophysiology of Tbx5-expressing CM in vivo, upon injury we created a tamoxifen-induced *Tbx5*^*CreERT2/+*^*/Rosa26R*^*eYFP/+*^*/Rosa26R*^*iDTR/+*^ transgene, where upon TAM administration, Tbx5-expressing cells will undergo apoptosis. It was observed that adult mice that received one dose of ISO and TAM had a 50% increased lethality, when compared to mice that only received TAM (Supplementary Fig. 6B). Histological examination 4 days-post injury showed severe LV and atrial injury in *Tbx5*^*CreERT2/+*^*/Rosa26R*^*eYFP/+*^*/Rosa26R*^*iDTR/+*^ mice that received ISO + TAM, while administration of TAM showed atrial injury only along with reduced ventricular damage, when compared to ISO + TAM (Supplementary Fig. 6C-I.). The cause of death was possibly due to reduced lung branching after the massive loss of alveolar Tbx5-expressing cells causing bronchiectasis (Supplementary Fig. 6C-II.). These findings place Tbx5 at a pivotal point for cardiac ventricular repair.

### The transcriptome of adult mammalian injury-induced YFP+ cells resembles that of developmentally earlier cardiac cells

**Fig. 6:**
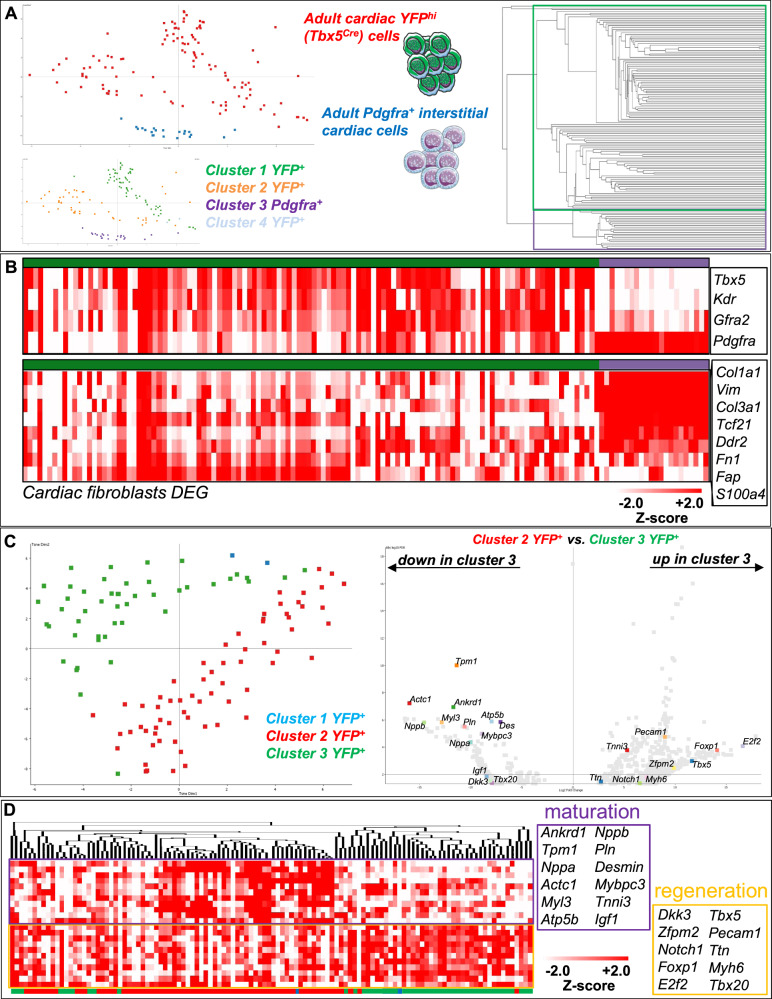
The transcriptome of YFP^+^ cell sub-clusters resembles that of CM precursors. **A** t-SNE dimensionality analysis and data store tree of EdgeR *p* < 0.05 after Benjamimi and Hochberg correction (8749 DEGs) of YFP^+^ (green) and Pdgfra^+^ (blue) interstitial adult heart cells. YFP^+^ cells could be divided into at least three sub-clusters. Single cells examined were obtained from 3 adult injured D7 hearts. **B** Heatmaps depicting expression of *Tbx5, Kdr, Gfra2 and Pdgfra* and cardiac fibroblast DEGs in YFP^+^ (green) and uninjured Pdgfra^+^ (purple) adult heart cells. **C** t-SNE dimensionality, and volcano plot of DEGs between the two major YFP^+^ sub-clusters (1091 DEGs). **D** Heatmap clustering analysis of CM-relevant genes within the DEG list that showed prominent expression differences between the two YFP^+^ sub-clusters.

**Fig. 7:**
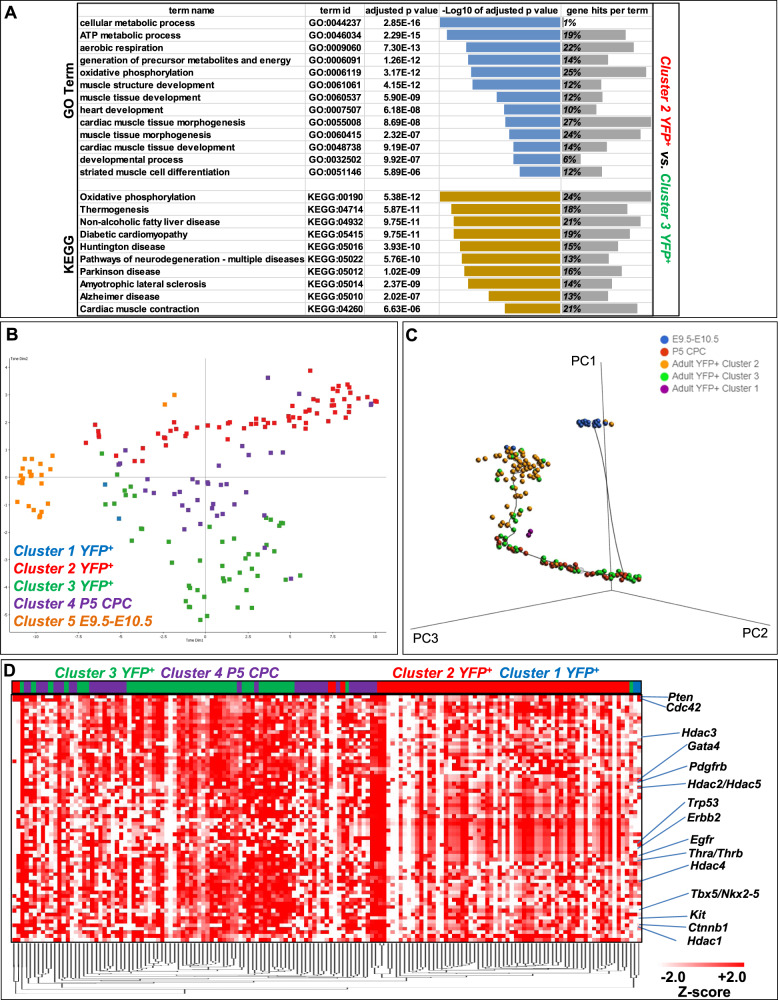
Developmental comparison of YFP^+^ CM. **A** Highlighted GO Biological Process and KEGG enrichment terms are shown. *p* < 0.025, FDR < 0.05 (Benjamimi and Hochberg correction). **B** t-SNE dimensionality analysis of all probes between YFP^+^ cell sub-clusters 1–3 (blue, red, green), P5 CPC (purple) and embryonic heart cells collected from E9.5 and E10.5 (orange). **C** Monocle 3 three-dimensional trajectory analysis of 177 cells with a starting node on E9.5–10.5 cardiac cell population (blue). P5 CPC (red), YFP^+^ sub-clusters 1–3 (purple, yellow, green, respectively). **D** Heatmap clustering analysis of STRING gene expression in YFP^+^ sub-clusters 1–3 (blue, red, green, respectively) and P5 CPC (purple) cells.

By employing single-cell RNA-seq (scRNA-seq) deep sequencing analysis on 116 YFP^+^-sorted cells and comparing them to Pdgfra^+^ uninjured interstitial cells, it was possible to confirm their distinct expression profile and reveal least two major YFP^+^ cell sub-clusters (Fig. 6A). Heatmap analysis on the FACS markers employed in this study and reference cardiac fibroblast genes^34^ confirmed their enrichment for *Tbx5, Gfra2, Kdr and Pdgfra*, and their underrepresentation, respectively in YFP^+^ cells, in relation to Pdgfra^+^ interstitial cardiac cells (Fig. 6B). In order to biologically identify and separate the two most prominent YFP^+^ cell sub-clusters we further statistically analyzed their DEGs (Fig. 6C). Gene Ontology (GO) and KEGG enrichment analysis revealed differences in oxidative phosphorylation, cardiac muscle development and morphogenesis, thermogenesis and hormonal responses (diabetic cardiomyopathy)^35^ as well as signaling related to central nervous system input (Fig. 7A).

The transcription factor Tbx5 has been shown to be expressed in embryonic cardiac cells that possess CM precursor properties, which can be faithfully recapitulated using our *Tbx5*^*CreERT2/CreERT2*^*;Rosa26R*^*eYFP/eYFP*^ transgene, in ex vivo settings (Supplementary Fig. 7A)^36^. To investigate the adult YFP^+^ cardiac cell population in-depth, a roadmap of single-cell RNA-seq transcriptomes was created from published single-cell RNA-seq in vivo E9.5-E10.5 cardiac progenitors^37^, as well as from CPC deriving from postnatal day 5, where cardiac regeneration is still achievable in mice (Fig. 7B). Clustering analysis underscored that adult YFP^+^ cells from the injured hearts were transcriptomically relevant to postnatal cardiac CPC populations, while more distant from early embryonic cardiac progenitors. Pseudotime developmental three-dimensional trajectory analysis confirmed the transcriptional similarity of the adult YFP^+^ cluster 3 to P5 postnatal CPC, yet away from the E9.5–10.5 cardiac cell population (Fig. 7C).

*Kmeans* STRING clustering analysis indicates that the Tbx5 transcriptional network is directly linked to thyroid hormonal responses (Supplementary Fig. 7B). Based on recent studies that involve thermogenesis and thyroid hormonal regulation in CM regeneration^35,38,39^, as well as our in silico meta-analysis data for thyroid hormone receptors binding to Tbx5, we interrogated the transcriptional profile of P5 CPC and YFP^+^ CMs in relation to the two major protein clusters, Tbx5 and Thyroid Hormone Receptors alpha and beta (Thrα/Thrβ) (Fig. 7). Heatmap clustering analysis indicated a similar expression pattern of Tbx5-related and Thrα/β-related genes in YFP^+^ CM cluster 3 and P5 CPC cells, when compared to YFP^+^ CM cluster 2.

## Discussion

The developing mammalian murine heart, initially, shares common progenitors with mesodermal progenitors of the cranial and paraxial mesoderm^40^, which have shown to give rise to muscle with regenerative capabilities^41,42^; the adult mammalian heart muscle lacks this property. Even if the injured heart has any substantial regenerative capacity, this is lost after the first week of age, in mice^43,44^. Recently, several studies have identified resident cells, which have been implicated in cardiomyocyte regeneration, yet this evidence has been heavily scrutinized. Eventually, it has been concluded that *de novo* cardiac stem cells are not present in the adult mammalian heart^45–48^.

The accumulated knowledge from embryonic cardiac development and CPCs has not been readily utilized in the adult regenerative field, with a few exceptions (Nkx2–5, Isl1) (For a review, please see ref. ^49^). In the current study, we employed a well-characterized transgenic mouse model capable of spatiotemporally lineage-tracing Tbx5^+^ cells during cardiac embryonic development^23^ and now, in adult mammalian hearts. The fact that this is a BAC insert, allows for investigating the spatiotemporal expression of Tbx5 in the adult heart, without inducing a cardiac phenotype^23^. Importantly, our transgene is capable of lineage-tracing ventricular Tbx5-expressing cardiac cells, which may be important for ventricle-specific repair/regeneration, in-line with recent published basic research and preclinical data^50–52^. In our study, Tbx5 re-activated CMs were located close to the lesion areas, but a few were also observed in non-injured areas of the adult heart. Recently, a major single-cell RNA-seq study using frozen human ischemic heart biopsies, designated *TBX5* overexpression in CM subpopulations as an important protective mechanism (pre-print server 10.1101/2021.06.23.449672).

In humans, TBX5 is expressed in both the embryonic and adult four-chambered heart, in yet unidentified cardiac cell populations^53^. In the adult mouse, Tbx5 has been shown to be primarily expressed in the atria but not in the ventricles, in the absence of injury, which we also observed^26^. We show that during chemical and I/R heart injuries, a CM-like ventricular subpopulation transiently re-activates Tbx5. Albeit present a month after injury, our quantification analysis did not show the formation of new in vivo YFP^+^-derived CM. In our experimental setting, we observed YFP^+^ CM displaying disrupted sarcomere and gap junctions peaking between 4 and 7 days after injury, with gap junctions being apparent a month after injury in some of those YFP^+^ CM. In vitro, YFP^+^ cells were capable of expressing CM markers but not smooth muscle or endothelial - like cells. Thus, it cannot be excluded that a low-level regeneration capability exists in the adult mammalian heart, albeit unable to compensate for the massive CM loss that occurs during a major heart incidence.

It is questionable whether a specific pre-designated CM subpopulation is primed for initiating CM replenishment and/or repair during injury, or instead this observation is a stochastic spatiotemporal event. We answer this question by underscoring the significance of Tbx5 in the adult heart; by omitting Tbx5-expressing cells *via* induced cell death, myocardial injury is aggravated. It is currently debatable whether an elusive regenerative mechanism in mammals is promoted by the presence of an endogenous pre-existing CM-like precursor population or a CM that directly re-enters the cell cycle^48^. Yet, these two possibilities are not mutually exclusive; they could imply that during myocardial insult, a subpopulation of CMs undergo dedifferentiation (i.e., transiently becoming CM precursors) through a process called epimorphosis, in order to proliferate and provide a source of new CMs. Indeed, a dedifferentiated regressive CM population that has regained a primitive phenotype, has been recently reported in adult murine and human injured hearts^54,55^. It has been documented that non-cardiomyocyte cell populations are actively proliferating in homeostatic postnatal^56^ and injured adult murine hearts^57^, showing no signs of apoptosis^58^. We confirmed this in our immunohistochemistry data, showing that proliferative interstitial cardiac cell populations were also present. Our results are indicative of an adult Tbx5-overexpressing ventricular cardiac cell that fits the profile of a potentially dedifferentiated CM-like precursor that fails to proliferate/re-differentiate, even if some cell cycle activators are expressed; they may form a septation-like zone around the injury site acting as guidepost cells^59^. In concert with our findings, a *Tbx5* mRNA expression burst has been documented in the border-zone of the heart between dead and viable CMs, termed border-zone CMs^54^. Interestingly, the authors of the same study identified that the cardiac-specific *natriuretic peptide precursor type A* (*Nppa*) gene is highly expressed in border-zone CMs; its expression is activated by the binding of Tbx5 and Nkx2–5 TFs in the *Nppa* promoter region^60^.

We reasoned that the Tbx5-lineage-tagged YFP^+^ adult cardiac population could be a potential cardiac precursor candidate, and for this, we employed knowledge gained from embryonic cardiac development, which can define and characterize that candidate^31^. The aforementioned CPC status of Gfra2^+^Pdgfra^+^Kdr^low/+^YFP^+^ cells was validated in vitro, in *Tbx5*^*CreERT2/CreERT2*^*;Rosa26R*^*eYFP/eYFP*^ mESC-derived CPCs prior to proceeding with our in situ investigation. We show here that an adult cardiac cell population exists in the injured mammalian heart that resembles the more well-defined embryonic cardiac precursors and postnatal precursors, in the surface marker expression levels and mRNA transcriptome, respectively. The presence of this triple surface-marker signature population observed only in the injured adult murine heart was also confirmed upon evaluation of metadata from previous scRNA-seq cardiac studies^61–63^. Of interest, not all of the adult Gfra2^+^Pdgfra^+^Kdr^low/+^ cardiac cells, expressed YFP. Yet, over 70% of the YFP^+^ cells did express Gfra2 and Pdgfra, while their Kdr expression profile was dynamic, similar to our mESC-derived CPC in vitro and embryonic data.

Interestingly, between P1 and P7, which is the only reported postnatal regenerative window of the murine heart, Gfra2^+^Pdgfra^+^Kdr^low/+^ cells were present in the absence of an insult. It remains to be seen if these cells are responsible for the aforementioned remuscularisation window, which has been recently shown to be affected by metabolic cues^64^. We therefore, set to explore through single-cell transcriptomic analysis how the adult YFP^+^ ventricular cells resemble early postnatal (P5) Gfra2^+^Pdgfra^+^Kdr^low/+^ and embryonic cardiac cells. Our findings reveal an adult cell population that transcriptomically resembles that of postnatal CM and less that of embryonic cardiac cells, a finding that is in line with adult limb regeneration studies conducted in amphibians^65^.

The apparent lack of an efficient in vivo regeneration upon mammalian adult cardiac injury may be attributed into two main distinct conditions; (i) an idle population capable of producing new CM, and (ii) a microenvironment that deters CM regeneration favouring resilience, thus increasing the chances of survival of the organism as a whole in the short-term (i.e., inducing sustainable fibrosis to avoid cardiac rupture)^66^. We report here that the first condition could be met, while the second one has been shown to be indeed a major hurdle for heart regeneration^35^. As such, endogenous CM-like precursors are present, yet unable to reach their true/full intrinsic regenerative potential.

Our studies raise the question of whether there is a distinct developmental origin of those adult Tbx5-expressing CM precursors of the LV, similar to what has been observed in the primitive streak^67^. Recently, Zhang et al. through an extensive *Mesp1*^*+*^-lineage tracing and scRNA-seq analyses, revealed that the FHF possess at least two early distinct cardiomyogenic progenitors, from which, a subset of the LV CMs, derived from Tbx5^+^ CPCs^68^. Here, we report that the YFP^+^ CM-like population is potentially responsive to CNS-derived signals, hinting towards a cardiac neural crest origin, as shown recently in the zebrafish^69^.

Bae et al. reported that malonate injections during MI, were capable of enhancing heart regeneration in adult mice^64^. The target CM subpopulation that drove the regeneration was not examined. It would be intriguing to assess whether the Tbx5^+^ lineage-traced population identified in our studies, is one of the plastic CM subpopulations affected by malonate and/or thyroxine, as also reported recently^35^.

One limitation of this study was the inability to collect YFP^+^ immediately after cardiac injury; this was due to the intracellular expression delay of the YFP protein (48 h after Tbx5 expression), which has been noted in our model and others’ (^23^ and references within). Therefore, although it is possible to assume that a similar cardiac regeneration window exists in the adult (as in neonates), where Tbx5 is transiently expressed, we were unable to collect a reliable number of valid YFP^+^ cells for further lineage-tracing analysis.

Another limitation is the absence of a reference point where adult mammalian CM regeneration is apparent. This hinders our ability to report that the Tbx5-expressing CM precursor cell population identified in the adult injured mammalian ventricles will eventually replenish the lost CMs. To overcome this, future studies will be also focusing on neonatal murine cardiac injuries, where established CM regeneration exists. Although this has been elegantly shown in adult non-mammalian organisms^21^, definitive experiments in mammals will allow us to confirm the potential of Tbx5-expressing CM precursors and definitively show that the latter are part of an idle mammalian cardiac regenerative program that is standing by.

In conclusion, this study reveals and characterizes an exclusive Tbx5-expressing ventricular CM-like precursor compartment identified following cardiac injury. As such, trending regenerative approaches can be tailored to target and trace the aforementioned cardiac cell population, which can be exploited to induce adult CM regeneration.

## Methods

### Animals

The BAC transgene *Tbx5*^*CreERT2*^ was constructed from the BAC clone RP23-267B15^70^ by replacement of exon 2 of Tbx5 with a CreERT2 cassette at the first methionine of the open reading frame in EL250 cells^23,71^. The *BAC-Tbx5*^*CreERT2*^ transgenes were crossed with *Rosa26R*^*eYFP/eYFP*^ transgenic mice (B6.129×1-Gt(ROSA)26Sortm1(EYFP)Cos/J) from Jackson laboratories stock# 006148, in order to produce *Tbx5*^*CreERT2*^*/Rosa26R*^*eYFP/eYFP*^ mice employed in this study. The *Tbx5*^*CreERT2/+*^*/Rosa26R*^*eYFP/+*^*/Rosa26R*^*iDTR/+*^ was created using the tamoxifen-induced *Rosa26R*^*iDTR/iDTR*^ transgene^72^ (Jackson laboratories stock# 007900) provided by the Klinakis lab (BRFAA).

All animal work has been approved by the BRFAA ethics committee and the Attica Veterinary Department (Animal Licence; 60876/23-1-20). All animals used were 2–3 months of age upon the time of ischemia/reperfusion (I/R) or isoproterenol administration experiments following relevant inclusion/exclusion guideline criteria^73^.

### Genotyping and PCR conditions

Genomic DNA extraction was performed from mouse tails with alkaline lysis (25 mM NaOH, 0.2 mM EDTA, pH = 12 for 1.5 h at 95 °C) and pH was neutralized with Tris-HCL (pH = 5) for 10 min RT. PCR conditions were as follow: initial denaturing step 3 min at 95 °C, 35 cycles (15 s at 95 °C, 15 s at 60 °C and 58 °C for Cre and eYFP primer pair respectively, 30 s at 72 °C) and final extension 5 min at 72 °C using KAPA Taq DNA polymerase (KAPA BIOSYSTEMS). PCR products were visualized on 2% agarose gels containing SYBR Safe DNA gel stain (Invitrogen). Primers used are;

CreRT2: F:5′- AGTTGCTTCAAAAATCCCTTCCAGGGCCCG -3′

R: 5′- AGCAATGCTGTTTCACTGGTTATGCGGCGG -3′

ROSA26 eYFP: F: 5′- GCGAAGAGTTTGTCCTCAACC -3′ R: 5′- AAAGTCGCTCTGAGTTGTTAT-3′

ROSA26 WT: 5′- GGAGCGGGAGAAATGGATATG- 3′ R: 5′- AAAGTCGCTCTGAGTTGTTAT- 3′

### Myocardial infraction models

I/R injury; MI was induced in 2–3 month-old mice by a 10 min transient ligation of the left anterior descending artery (LAD) based on an established protocol^74^ with some modifications. Briefly, mice were anesthetized by intraperitoneal injection with a combination of ketamine and xylazine (0.01 ml/g, final concentrations of ketamine and xylazine, 10 and 2 mg/ml, respectively). Anesthetic depth was evaluated by the loss of pedal reflex to toe-pinch stimulus and breathing rate. A thoracotomy was then performed between the fourth and fifth ribs, and the pericardium was carefully retracted to visualize the left anterior descending coronary, which was ligated using a 7-0 Prolene monofilament polypropylene suture placed 3 mm below the tip of the left auricle. After the ischemic period, the ligature was released, allowing reperfusion of the myocardium. Hearts were obtained 5 days after I/R (*N* = 8).

Isoproterenol-induced cardiac infraction; Adult two-three month-old *Tbx5*^*CreERT2*^*/Rosa26R*^*eYFP/eYFP*^*, Tbx5*^*CreERT2/+*^*/Rosa26R*^*eYFP/+*^*/Rosa26R*^*iDTR/+*^ and control littermates were injected with isoproterenol (ISO, 20 mg/kg per day intraperitoneally, Sigma-Aldrich; St. Louis, I6504) once daily for two consecutive days^75–78^. Hearts were obtained and examined 2, 4, 6, 7 and 30 days after the last ISO injection (*N* = 60).

Tamoxifen diluted in peanut oil (Sigma-Aldrich; St. Louis, P2144) was administrated on days 1 and 2 to all animals, at a final concentration of 0.8 mg/10 g of body weight, by oral gavage (*N* = 90).

### Single cell RNA-Seq library preparation and deep sequencing

The Fluidigm C1 system was used to prepare single cells for RNA-Seq. RNA-Seq-IFCs were selected to capture all major cell populations from all cell size ranges observed using IFCs which capture cells of different sizes: 5–10 μM (embryonic), 10–17 μM (embryonic, neonatal), 17–25 μM (>3 weeks of age). No batch effects were observed between chips of the same size. Onboard cell lysis, reverse transcription and cDNA synthesis were performed using the SMART-Seq v4 Ultra Low RNA Kit for the Fluidigm C1 System (Takara) reagents, following the manufacturer’s protocol. The resulting cDNAs from individual cells were used for the construction of NGS libraries with the Nextera XT DNA sample preparation kit (Illumina). Libraries were pooled, quantified with qubit HS DNA spectrophotometer and quality control was performed with the Agilent Bioanalyzer HS DNA kit. Approximately 1 Million 2 × 150bp Paired End Reads were generated for each single-cell RNA-Seq library in Illumina NovaSeq system following the manufacturer’s standard protocol. Count data were normalized to counts per million and transformed to Log2(CPM + 1). Single-cell libraries with >500,000 reads and <5% in mitochondrial genes were used for further analysis.

### Single-cell cDNA expression profiling

Embryonic murine heart cells were used from our previous studies and other research groups^23,37^ for the purpose of comparing our in vitro embryonic and adult cardiac cell CPC transcriptomes. Embryonic FACS-sorted CPC on days 7 and 9 in vitro differentiation and adult CPC were collected *via* FACS sorting and further analyzed using the Fluidigm C1 machine and workflow according to the manufacturer’s protocol. We examined a total of 20 cells derived from embryonic heart between E9.5-E10.5, 76 cells derived from P5 CPC, 22 Pdgfra^+^ interstitial adult cardiac cells and 240 YFP^+^ cells from D7 injured ventricles.

Sequence data have already been submitted to NCBI Gene Expression Omnibus (GEO, http://www.ncbi.nlm.nih.gov/geo) under the accession numbers GSE63796 and at CNCB with accession number PRJCA013789.

### NGS data analysis pipeline

FASTQ data were quality tested and aligned to the murine genome using the online www.useGalaxy.eu platform. BAM files were created using the gapped-read mapper RNA-STAR alignment software using the mm10 murine primary assembly^79^. RNA-STAR parameters are depicted in Supplementary Table 1. BAM files were further sorted using Samtools sort^80^. Aligned and sorted BAM files were further analyzed on SeqMonk 1.48.0 software as shown previously^81^ (Supplementary Fig. 8 and Supplementary Data 1). For Gene Ontology (GO) and KEGG downstream analysis, toppgene online tool^82^, as well as cytoscape and ClueGo^83^ software, and STRING^TM^ online tool were employed. For single-cell trajectory analysis, Partek Flow^TM^ was employed, deploying the integrated Monocle 3 software.

### Cardiac differentiation of murine ES cells

Cardiac differentiation of mouse ES cells (both BAC-*Tbx5*^*Cre*^*/R26R*^*eYFP/eYFP*^ and BAC-*Tbx5*^*Cre*^*/Rosa26R*^*eYFP/eYFP*^*/Tbx5*^*KO/KO*^ cell lines) was induced via monolayer and embryoid body conditions as previously described^23,24^. In brief, undifferentiated colonies were passaged for cell counting and re-seeded onto 24-well laminin-coated plates (5 μg/ml) (Biolaminin LN521, BioLamina) pre-incubated with laminin for 2 h at 37 °C under 5% CO_2_, at a cell density of 120,000 cells/well. Cells were cultured in ESGRO Complete medium plus LIF (Millipore 2i Medium Kit) for a day further. For three-step differentiation, cells were incubated for 1 day in IMDM/Ham’s F12 (Invitrogen) supplemented with N2 and B27 supplements (Gibco), 10% (stock) bovine serum albumin (Sigma), 2 mM L-glutamine (Gibco), penicillin-streptomycin (Gibco), 0.5 mM ascorbic acid (Sigma), and 0.45 mM monothioglycerol (MTG, Sigma). For mesodermal induction and patterning, cells were exposed for 2 days to human Activin A (8 ng/ml, R&D Systems) and human bone morphogenetic protein 4 (hBMP4, 1.5 ng/ml; R&D Systems) together with human vascular endothelial growth factor (hVEGF, 5 ng/ml; R&D Systems). Cardiac specification was induced by exposure of cells to StemPro-34 SF medium (Gibco) supplemented with 2 mM L-glutamine, 0.5 mM ascorbic acid, human VEGF (5 ng/ml), human basic fibroblast growth factor (bFGF, 10 ng/ml, R&D Systems), and human fibroblast growth factor 10 (FGF10, 50 ng/ml, R&D Systems). The medium was changed every other day. All media were prepared under a sterile hood (Class 2), filtered through a Millipore Stericup 0.22 mm filtration system and stored at 4 °C. Upon medium exchange, cells were washed twice with phosphate buffered saline (PBS, Gibco). Medium was changed every other day, and cells were analyzed on various designated days. EBs (3000 cells per 30 μl in each drop) were dissociated for medium changes. 4-hydroxytamoxifen (4OH-TAM) administration (500 nM) began on day 4 of differentiation and was renewed along with medium changes every other day.

### Cardiac single-cell suspension preparation

Adult hearts (and parts of) were collected and harvested on days 2, 4 and 7 after MI and single-cell suspensions were prepared immediately before analysis by flow cytometry as previously described^84^. In brief, postnatal and adult hearts were minced and digested in 2.5 mg/mL collagenase D (Roche), 0.25 mg/mL DNase I (Roche) and 0.05% Trypsin-EDTA solution in RPMI (Sigma) incubated for 45 min at 37 °C accompanied with constant pipetting for mechanical separation. Digested samples were passed through a 70 μm Nylon cell strainer, washed and suspended in Hank’s Balanced Salt Solution (HBSS, Gibco) with 3% FBS and 0.03 mM EDTA (FACS buffer) for staining.

### Flow cytometry of cultured cells

Murine ES-derived CPC were analyzed for the presence of appropriate markers on designated days of mesodermal and cardiac differentiation with the use of an ARIA II Analyzer (BD Biosciences) and FACSDiva 7.0 software as previously described^24^. In brief, cultured cells were treated with 0.05% trypsin/EDTA (Gibco) for 5 min at 37 °C under 5% CO_2_. Cells were labeled with the following antibodies: anti-human/mouse GFRA2 Polyclonal Goat IgG (R&D Systems, Cat no. AF429), rat monoclonal anti-PDGFR alpha antibody conjugated with PE (Abcam, APA5, Cat no. ab93531), donkey polyclonal anti-goat IgG Alexa 405 conjugated with UV (Abcam, Cat no. ab175664), rat monoclonal IgG2b anti-mouse KDR-Alexa647 conjugated with APC (BioLegend, Cat no. 121910). 7-AAD (BioLegend, Cat no. 420404) was used as a viability marker. Sca-1 (Biolegend Cat no. 108127), c-Kit (Biolegend, Cat no. 105813), CD31 (Biolegend, Cat no. 102524). All abs, were used at 1/100 dilution. Flow Cytometry data analysis was performed using FlowJo^TM^ V10.

### FACS-sorted cell culture conditions

Acquired sorted cells were harvested in 50% FBS in PBS and centrifuged for 20 min at 4 °C. After centrifuging, cells were seeded onto 96-well laminin-coated plates and cultured in 20% FBS/DMEM (Gibco) with penicillin-streptomycin (Gibco). Medium was changed every 3 days and cells were fixed on designated days for further analysis.

### Cell culture immunofluorescence staining

Cultured cells were fixed with pre-warmed 4% paraformaldehyde (PFA) for 10 min at room temperature. Fixed cells were washed three times for 5 min in PBS, and then nonspecific antibody binding sites were blocked with blocking buffer 1% BSA/0.2% Triton X-100 in PBS for 30 min at RT. After that cells were incubated with primary antibodies in blocking buffer overnight at 4 °C. Primary antibodies: Cardiac troponin T (cTnT, 1/100, mouse monoclonal, Abcam, Cat no. ab28364), alpha smooth muscle actin (aSMA) (1/100, rabbit polyclonal, Abcam, Cat no. ab6046) and CD31 (1/25, Rabbit polyclonal, Abcam, Cat no. ab38689). For enhancing the endogenous YFP signal, in ICC, we used anti-GFP FITC-conjugated (1/100, goat polyclonal, Abcam, Cat no. ab6662).

After rinsing 3 times for 5 min with PBS, cells were incubated with secondary antibodies for 1 h at RT: Alexa Fluor 555 Goat anti-mouse IgG, Alexa Fluor 647 Goat anti-rabbit IgG. Again, cells were washed three times for 5 min in PBS and then mounted with DAPI mounting medium (Fluoroshield with DAPI, Sigma-Aldrich). Images were acquired with an inverted Leica DMΙRΕ2 microscope and a Hamamatsu Camera ORCA Flash 4.0 LT.

### Heart tissue sectioning and staining

Adult murine hearts were isolated and fixed in 4% PFA in PBS and embedded in paraffin, sectioned transversely at 5 μm thick and mounted onto slides. The sections underwent deparaffinization with xylene and a decreasing ethanol gradient and were routinely stained with Haematoxylin and Eosin (H&E). Masson’s Trichrome staining was used in paraffin sections to identify collagen fibers in 1-month damaged murine hearts. Upon deparaffinization, staining were used as followed: Hematoxyline Harris 1 min, Red of Mallory 3 min (Fuchsin acid, Sigma), Molybdophosphoric acid 1% 2 min (dodeca-Molybdophosphoric acid, Vyzas), Methyl blue 1 min (Sigma). Between different stains slides were washed with dH2O. Lastly, slides were dehydrated with: 100% EtOH (1st) 1 min, 2nd EtOH 2 min, acidified EtOH 2 min, 1st Xylene 4 min, and 2nd Xylene 4 min.

For immunohistochemistry, acquired adult murine hearts (and parts of) were perfused and fixed with 4% PFA in PBS, for 2 h at 4 °C. Then, they were transferred in 30% sucrose in PBS, at 4 °C overnight. The next day they were embedded in OCT compound (VWR) and 16 μm thick cryosections were prepared. Cryosections were post-fixed with pre-warmed 4% PFA in PBS for 15 min, and rinsed in PBS. Sections were blocked with 2% BSA (fraction V)/ 10% FBS/0.05% Tween 20 in PBS, at room temperature (RT) for 1.5 h incubated with primary antibodies in the blocking buffer at 4 °C overnight. Primary antibodies used were: MF20 (mouse monoclonal, 1/100, Developmental Biology Hybridoma Bank), Tbx5 (rabbit polyclonal, 1/100, Sigma), GFRA2 (chicken polyclonal, 1/500, Antibodies-online, ABIN1450225), Connexin 43 (cat no C6219-.2 ML, rabbit polyclonal, 1/2000, Sigma), α-actinin (A7811, clone EA-53, mouse monoclonal, 1/500, Sigma), Ki67 (ab15580, rabbit polyclonal, 1:100, Abcam). For enhancing the endogenous YFP signal in IHC, we used anti-GFP (chicken polyclonal, 1/1000, Abcam). Border and Injury zones were clarified as shown previously^27,28^.

After washing 3 times with 0.5% Triton X-100 in PBS (PBST), samples were incubated with the secondary antibody in the blocking buffer for 1 h at RT. Secondary antibodies used were as follows: Alexa Fluor 555 Goat Anti-mouse IgG, Alexa Fluor 647 Goat Anti-rabbit IgG, Alexa Fluor 488-conjugated Goat Anti-chicken (All from Invitrogen at concentration of 1/1000). After washing 3 times with PBST sections were mounted with DAPI. Fluorescent images were captured using an upright Leica DMRA2 fluorescence microscope and a Hamamatsu ORCA-Flash 4.0 V2.

### Reverse transcription and quantitative real-time PCR analysis

Hearts were collected on days 2, 4 and 7 after injury, and total RNA was extracted using TRIzol Reagent (Sigma-Aldrich, T9424) and chloroform (AppliChem). For each specimen 500 ng of total RNA was reversed transcribed into cDNA using PrimeScript RT reagent kit (TaKaRa RR037a) and oligo dT primers according to the manufacturer’s protocol. Prior to cDNA synthesis, samples were subjected to TURBO^TM^ DNase (Invitrogen) treatment for 1 h at 37 °C and 10 min at 75 °C. Quantitate PCR was conducted using KAPA SYBR FAST Master Mix (Sigma-Aldrich, KK4611) on a Roche Lightcycler 96 (Roche Life Science). Cycling conditions were as follows: 2 min at 50 °C and 10 min at 95 °C (Pre-incubation) followed by two-step PCR for 40 cycles of 15 s at 95 °C and 60 s at 60 °C. Expression levels were calculated using the comparative CT method and calculated 2^−ΔΔCt^ values are presented. Values for specific genes were normalized to *GAPDH* as a constitutively expressed internal control. Primers used are;

*eYFP:* F: 5′-ACGTAAACGGCCACAAGTTC-3′ R: 5′-AAGTCGTGCTGCTTCATGTG-3′

*Tbx5:* F: 5′-CTCCCAGCAAGTCTCCATCA-3′ R: 5′-GGCCAGTCACCTTCACTTTG-3′

*Gapdh:* F: 5′-AGGTCGGTGTGAACGGATTTG-3′ R: 5′-TGTAGACCATGTAGTTGAGGTCA-3′

### Statistics

Flow cytometric data are presented as mean ± SD. Statistical analysis on FACS data was performed using ANOVA T-test using Mann–Whitney or Bonferroni post hoc test, where appropriate (*p* < 0.05). Statistical analyses were calculated using GraphPad Prism 5. Single-cell RNA-seq data statistical analysis was initially performed using *p*-value (<0.05) and EdgeR after Benjamini and Hochberg^85^ for obtaining DE genes (DEGs). Downstream analysis involved False Discovery Analysis (FDR) based on Benjamini and Hochberg^85^. All statistical analyses were performed using GraphPad Prism, with the threshold for significance set at *P* < 0.05.

### Reporting summary

Further information on research design is available in the Nature Research Reporting Summary linked to this article.

## Supplementary information

Supplementary Information

Supplementary Data 1

Reporting Summary

Video accompanying supplementary Figure 7A

Video accompanying supplementary Figure 7A

## Data Availability

The data that support the findings of this study are available from the corresponding author upon reasonable request. Sequencing data are deposited in NCBI Gene Expression Omnibus (GEO, http://www.ncbi.nlm.nih.gov/geo) under the accession numbers GSE63796 and at CNCB with accession number PRJCA013789.

## References

[1] Virani SS (2020). Heart disease and stroke statistics-2020 update: a report from the American Heart Association. Circulation.

[2] Evans SM, Yelon D, Conlon FL, Kirby ML (2010). Myocardial lineage development. Circ. Res..

[3] Rana MS, Christoffels VM, Moorman AF (2013). A molecular and genetic outline of cardiac morphogenesis. Acta Physiol..

[4] Downs KM, Davies T (1993). Staging of gastrulating mouse embryos by morphological landmarks in the dissecting microscope. Development.

[5] Tam PP, Behringer RR (1997). Mouse gastrulation: the formation of a mammalian body plan. Mech. Dev..

[6] Buckingham M, Meilhac S, Zaffran S (2005). Building the mammalian heart from two sources of myocardial cells. Nat. Rev. Genet..

[7] Ma Q, Zhou B, Pu WT (2008). Reassessment of Isl1 and Nkx2-5 cardiac fate maps using a Gata4-based reporter of Cre activity. Dev. Biol..

[8] Hsieh PC (2007). Evidence from a genetic fate-mapping study that stem cells refresh adult mammalian cardiomyocytes after injury. Nat. Med..

[9] Bergmann O (2009). Evidence for cardiomyocyte renewal in humans. Science.

[10] Mollova M (2013). Cardiomyocyte proliferation contributes to heart growth in young humans. Proc. Natl Acad. Sci. USA.

[11] Ali SR (2014). Existing cardiomyocytes generate cardiomyocytes at a low rate after birth in mice. Proc. Natl Acad. Sci. USA.

[12] Malliaras K (2013). Cardiomyocyte proliferation and progenitor cell recruitment underlie therapeutic regeneration after myocardial infarction in the adult mouse heart. EMBO Mol. Med..

[13] Senyo SE (2013). Mammalian heart renewal by pre-existing cardiomyocytes. Nature.

[14] Steimle JD, Moskowitz IP (2017). TBX5: a key regulator of heart development. Curr. Top. Dev. Biol..

[15] Bruneau BG (1999). Chamber-specific cardiac expression of Tbx5 and heart defects in Holt-Oram syndrome. Dev. Biol..

[16] Jia Y, Chang Y, Guo Z, Li H (2019). Transcription factor Tbx5 promotes cardiomyogenic differentiation of cardiac fibroblasts treated with 5-azacytidine. J. Cell Biochem..

[17] Kathiriya IS (2020). Modeling human TBX5 haploinsufficiency predicts regulatory networks for congenital heart disease. Dev. Cell.

[18] Inagawa, K. et al. Induction of cardiomyocyte-like cells in infarct hearts by gene transfer of Gata4, Mef2c, and Tbx5. *Circ. Res*. 10.1161/CIRCRESAHA.112.271148 (2012). 10.1161/CIRCRESAHA.112.271148 22931955

[19] McDonnell TJ, Oberpriller JO (1984). The response of the atrium to direct mechanical wounding in the adult heart of the newt, Notophthalmus viridescens. An electron-microscopic and autoradiographic study. Cell Tissue Res..

[20] Grajevskaja V, Camerota D, Bellipanni G, Balciuniene J, Balciunas D (2018). Analysis of a conditional gene trap reveals that tbx5a is required for heart regeneration in zebrafish. PLoS One.

[21] Sanchez-Iranzo H (2018). Tbx5a lineage tracing shows cardiomyocyte plasticity during zebrafish heart regeneration. Nat. Commun..

[22] Wu CC (2016). Spatially resolved genome-wide transcriptional profiling identifies BMP signaling as essential regulator of zebrafish cardiomyocyte regeneration. Dev. Cell.

[23] Kokkinopoulos I (2015). Single-cell expression profiling reveals a dynamic state of cardiac precursor cells in the early mouse embryo. PLoS One.

[24] Kokkinopoulos I (2016). Cardiomyocyte differentiation from mouse embryonic stem cells using a simple and defined protocol. Dev. Dyn..

[25] Cowled, P. & Fitridge, R. *Mechanisms of Vascular Disease: A Reference Book for Vascular Specialists* (eds Fitridge, R. & Thompson, M.). (University of Adelaide Press, 2011). 30484990

[26] Arnolds DE (2012). TBX5 drives Scn5a expression to regulate cardiac conduction system function. J. Clin. Invest..

[27] Forte E (2021). Type 2 MI induced by a single high dose of isoproterenol in C57BL/6J mice triggers a persistent adaptive immune response against the heart. J. Cell Mol. Med..

[28] Mielcarek M (2014). The Huntington’s disease-related cardiomyopathy prevents a hypertrophic response in the R6/2 mouse model. PLoS One.

[29] Huang W (2018). Loss of microRNA-128 promotes cardiomyocyte proliferation and heart regeneration. Nat. Commun..

[30] Kattman SJ, Huber TL, Keller GM (2006). Multipotent flk-1+ cardiovascular progenitor cells give rise to the cardiomyocyte, endothelial, and vascular smooth muscle lineages. Dev. Cell.

[31] Ishida H (2016). GFRA2 identifies cardiac progenitors and mediates cardiomyocyte differentiation in a RET-independent signaling pathway. Cell Rep..

[32] Jesty SA (2012). c-kit+ precursors support postinfarction myogenesis in the neonatal, but not adult, heart. Proc. Natl Acad. Sci. USA.

[33] Patterson M (2017). Frequency of mononuclear diploid cardiomyocytes underlies natural variation in heart regeneration. Nat. Genet..

[34] Ivey MJ, Tallquist MD (2016). Defining the cardiac fibroblast. Circ. J..

[35] Hirose K (2019). Evidence for hormonal control of heart regenerative capacity during endothermy acquisition. Science.

[36] Bruneau BG (2013). Signaling and transcriptional networks in heart development and regeneration. Cold Spring Harb. Perspect. Biol..

[37] Li G (2016). Transcriptomic profiling maps anatomically patterned subpopulations among single embryonic cardiac cells. Dev. Cell.

[38] Pantos C, Mourouzis I (2018). Thyroid hormone receptor alpha1 as a novel therapeutic target for tissue repair. Ann. Transl. Med..

[39] Pantos C, Mourouzis I (2015). Translating thyroid hormone effects into clinical practice: the relevance of thyroid hormone receptor alpha1 in cardiac repair. Heart Fail Rev..

[40] Tyser RCV (2021). Characterization of a common progenitor pool of the epicardium and myocardium. Science.

[41] De Micheli AJ (2020). Single-cell analysis of the muscle stem cell hierarchy identifies heterotypic communication signals involved in skeletal muscle regeneration. Cell Rep..

[42] Dell’Orso, S. et al. Single cell analysis of adult mouse skeletal muscle stem cells in homeostatic and regenerative conditions. *Development*10.1242/dev.174177 (2019). 10.1242/dev.174177 PMC6602351 30890574

[43] Mahmoud AI (2013). Meis1 regulates postnatal cardiomyocyte cell cycle arrest. Nature.

[44] Porrello ER (2011). Transient regenerative potential of the neonatal mouse heart. Science.

[45] Vicinanza C (2018). Kit(cre) knock-in mice fail to fate-map cardiac stem cells. Nature.

[46] van Berlo JH (2014). c-kit+ cells minimally contribute cardiomyocytes to the heart. Nature.

[47] Ellison GM (2013). Adult c-kit(pos) cardiac stem cells are necessary and sufficient for functional cardiac regeneration and repair. Cell.

[48] Li Y (2018). Genetic lineage tracing of nonmyocyte population by dual recombinases. Circulation.

[49] Le T, Chong J (2016). Cardiac progenitor cells for heart repair. Cell Death Discov..

[50] Jiang, L. et al. CRISPR activation of endogenous genes reprogramsfibroblasts into cardiovascular progenitorcells for myocardial infarction therapy. *Mol Ther*. 10.1016/j.ymthe.2021.10.015 (2021).

[51] Isomi M (2021). Overexpression of Gata4, Mef2c, and Tbx5 generates induced cardiomyocytes via direct reprogramming and rare fusion in the heart. Circulation.

[52] Rathjens FS (2021). Preclinical evidence for the therapeutic value of TBX5 normalization in arrhythmia control. Cardiovasc. Res..

[53] Hatcher CJ, Goldstein MM, Mah CS, Delia CS, Basson CT (2000). Identification and localization of TBX5 transcription factor during human cardiac morphogenesis. Dev. Dyn..

[54] van Duijvenboden K (2019). Conserved NPPB+ border zone switches from MEF2- to AP-1-driven gene program. Circulation.

[55] Zhang Y (2019). Single-cell imaging and transcriptomic analyses of endogenous cardiomyocyte dedifferentiation and cycling. Cell Discov..

[56] O’Meara CC (2015). Transcriptional reversion of cardiac myocyte fate during mammalian cardiac regeneration. Circ. Res..

[57] Kretzschmar, K. et al. Profiling proliferative cells and their progeny in damaged murine hearts. *Proc. Natl Acad. Sci. USA*10.1073/pnas.1805829115 (2018). 10.1073/pnas.1805829115 PMC6310797 30530645

[58] Dispersyn GD (2002). Dissociation of cardiomyocyte apoptosis and dedifferentiation in infarct border zones. Eur. Heart J..

[59] Waldron L (2016). The cardiac TBX5 interactome reveals a chromatin remodeling network essential for cardiac septation. Dev. Cell.

[60] Hiroi Y (2001). Tbx5 associates with Nkx2-5 and synergistically promotes cardiomyocyte differentiation. Nat. Genet..

[61] McLellan MA (2020). High-resolution transcriptomic profiling of the heart during chronic stress reveals cellular drivers of cardiac fibrosis and hypertrophy. Circulation.

[62] Farbehi, N. et al. Single-cell expression profiling reveals dynamic flux of cardiac stromal, vascular and immune cells in health and injury. *eLife*10.7554/eLife.43882 (2019). 10.7554/eLife.43882 PMC6459677 30912746

[63] Skelly DA (2018). Single-cell transcriptional profiling reveals cellular diversity and intercommunication in the mouse heart. Cell Rep..

[64] Bae J (2021). Malonate promotes adult cardiomyocyte proliferation and heart regeneration. Circulation.

[65] Lin TY (2021). Fibroblast dedifferentiation as a determinant of successful regeneration. Dev. Cell.

[66] Notari M (2018). The local microenvironment limits the regenerative potential of the mouse neonatal heart. Sci. Adv..

[67] Ivanovitch K (2021). Ventricular, atrial, and outflow tract heart progenitors arise from spatially and molecularly distinct regions of the primitive streak. PLoS Biol..

[68] Zhang Q (2021). Unveiling complexity and multipotentiality of early heart fields. Circ. Res..

[69] Tang W, Martik ML, Li Y, Bronner ME (2019). Cardiac neural crest contributes to cardiomyocytes in amniotes and heart regeneration in zebrafish. eLife.

[70] Smemo S (2012). Regulatory variation in a TBX5 enhancer leads to isolated congenital heart disease. Hum. Mol. Genet..

[71] Uehara M, Yashiro K, Takaoka K, Yamamoto M, Hamada H (2009). Removal of maternal retinoic acid by embryonic CYP26 is required for correct Nodal expression during early embryonic patterning. Genes Dev..

[72] Buch T (2005). A Cre-inducible diphtheria toxin receptor mediates cell lineage ablation after toxin administration. Nat. Methods.

[73] Lindsey ML (2021). Guidelines for in vivo mouse models of myocardial infarction. Am. J. Physiol. Heart Circ. Physiol..

[74] Chatzianastasiou A (2016). Cardioprotection by H2S donors: nitric oxide-dependent and independent mechanisms. J. Pharm. Exp. Ther..

[75] Brooks WW, Conrad CH (2009). Isoproterenol-induced myocardial injury and diastolic dysfunction in mice: structural and functional correlates. Comp. Med..

[76] Song Y (2012). Cardiac ankyrin repeat protein attenuates cardiac hypertrophy by inhibition of ERK1/2 and TGF-beta signaling pathways. PLoS One.

[77] Oudit GY (2003). Phosphoinositide 3-kinase gamma-deficient mice are protected from isoproterenol-induced heart failure. Circulation.

[78] Miyao N (2020). TBX5 R264K acts as a modifier to develop dilated cardiomyopathy in mice independently of T-box pathway. PLoS One.

[79] Dobin A (2013). STAR: ultrafast universal RNA-seq aligner. Bioinformatics.

[80] Li H (2009). The sequence alignment/map format and SAMtools. Bioinformatics.

[81] Kokkinopoulos I (2021). Patrolling human SLE haematopoietic progenitors demonstrate enhanced extramedullary colonisation; implications for peripheral tissue injury. Sci. Rep..

[82] Chen J, Bardes EE, Aronow BJ, Jegga AG (2009). ToppGene Suite for gene list enrichment analysis and candidate gene prioritization. Nucleic Acids Res..

[83] Bindea G (2009). ClueGO: a Cytoscape plug-in to decipher functionally grouped gene ontology and pathway annotation networks. Bioinformatics.

[84] Pinto AR (2016). Revisiting cardiac cellular composition. Circ. Res..

[85] Benjamini Y, Hochberg Y (1995). Controlling the false discovery rate: a practical and powerful approach to multiple testing. J. R. Stat. Soc. Ser. B (Methodol.).

