# Supplementary material for: Return of the Tbx5; lineage-tracing reveals ventricular cardiomyocyte-like precursors in the injured adult mammalian heart
Source: NPJ Regen Med. 2023 Mar 3;8:13. doi: 10.1038/s41536-023-00280-9 (PMC9984483; doi:10.1038/s41536-023-00280-9)
Supplement: Supplementary file 1 — Supplementary Information [file 41536_2023_280_MOESM1_ESM.pdf]

Supplementary Figure 1 **Siatra *et al.***

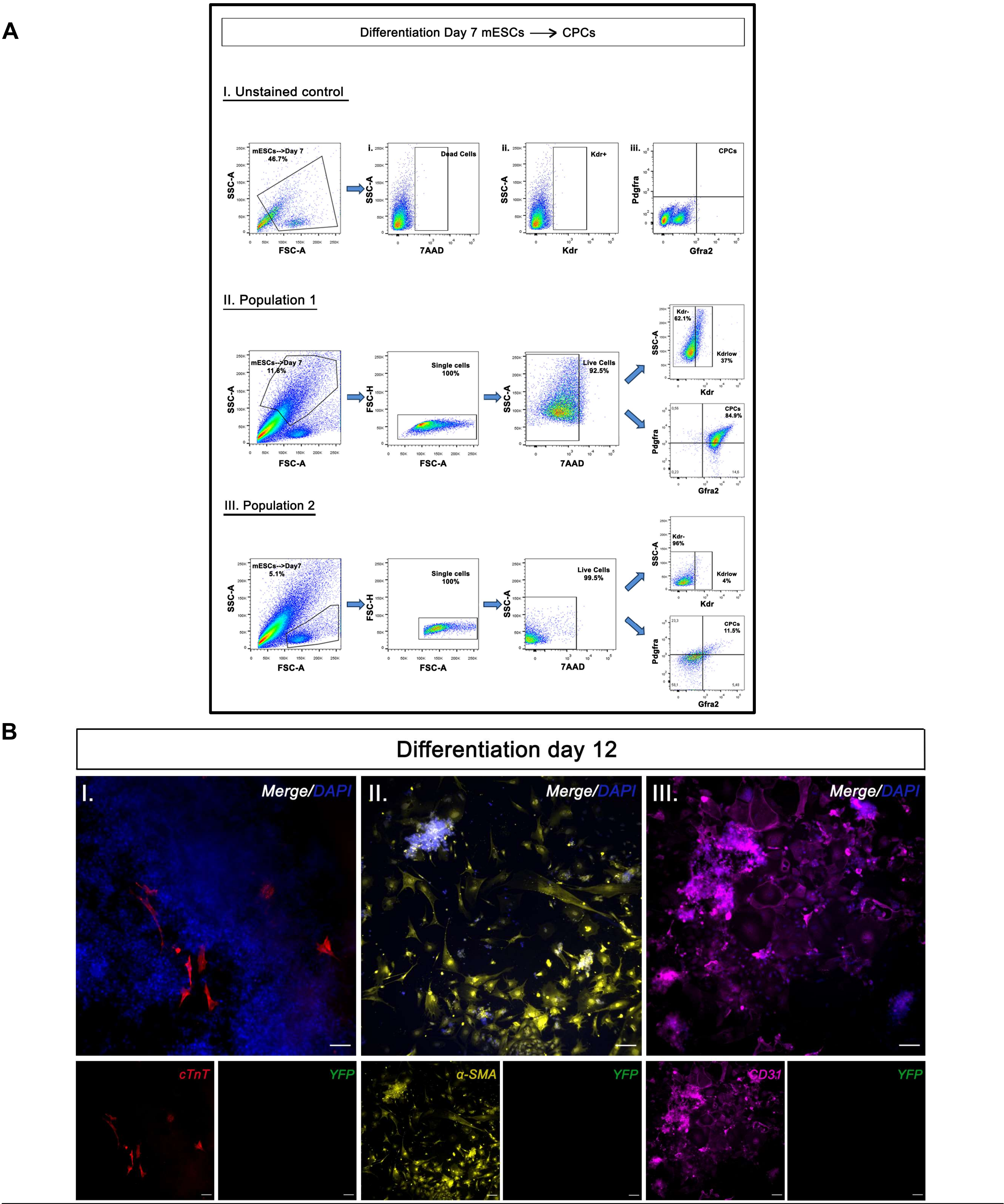

Supplementary Figure 1. (**A**) Gating strategy for acquiring triple positive cells from *in vitro* mESC in CM differentiation conditions, 7 days after 3i medium removal. (**B**) Immunocytochemical analysis of $\textit{Tbx5}^{\textit{Cre}};\textit{R26R}^{\textit{eYFP/eYFP}}$ mESC-derived differentiated cells in suboptimal CM differentiation conditions, after 12 days *in vitro*. Scale bar = 100μm.

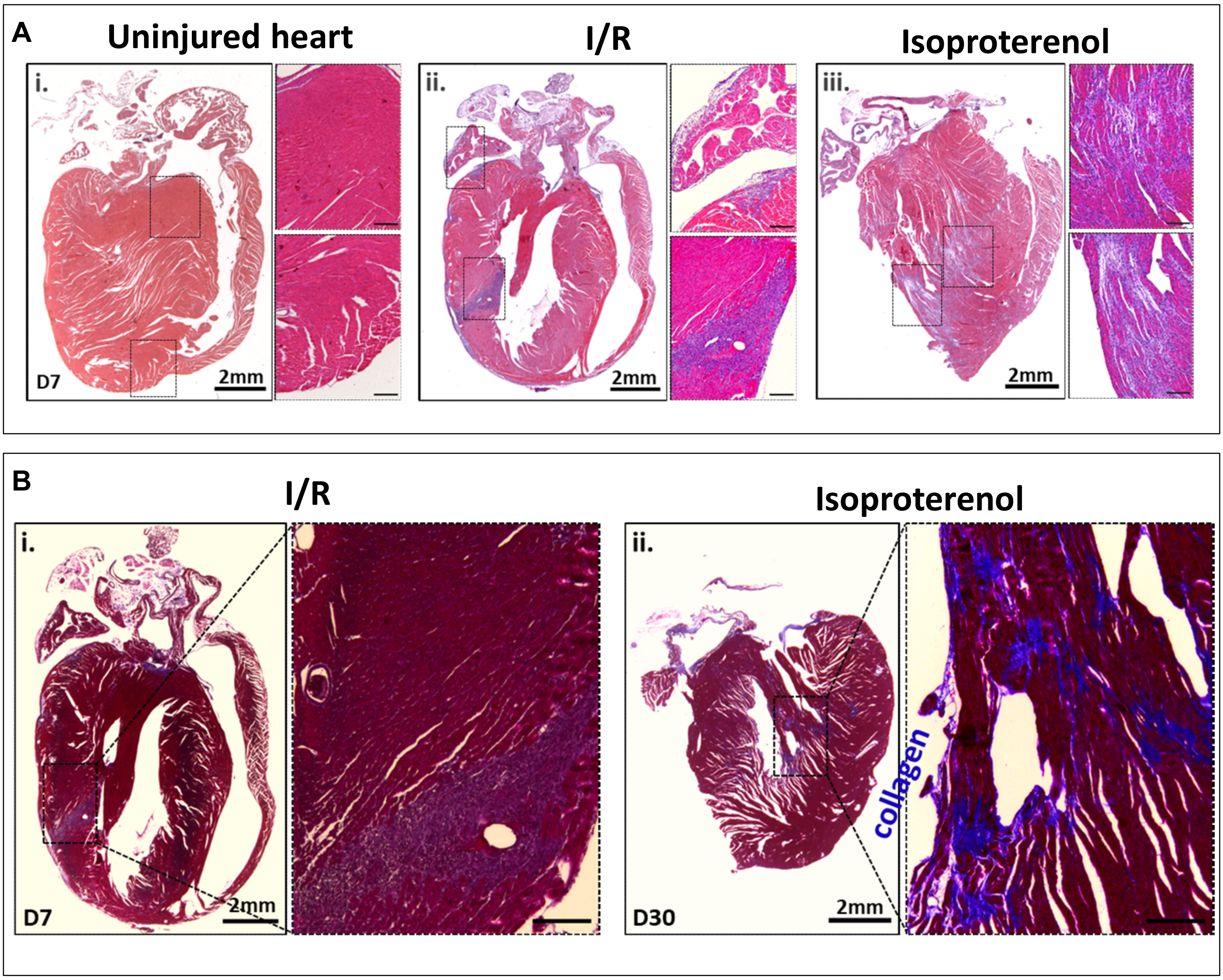

Supplementary Figure 2. (**A**) Haematoxylin and Eosin staining as well as whole heart photographs confirmed non-fatal, yet substantial, heart injury in the left ventricle, when compared to non-MI adult hearts. (**B**) Masson's staining, of collagen deposition at D7 and D30 after injury. N=1-3 per condition. Scale bar 100μm.

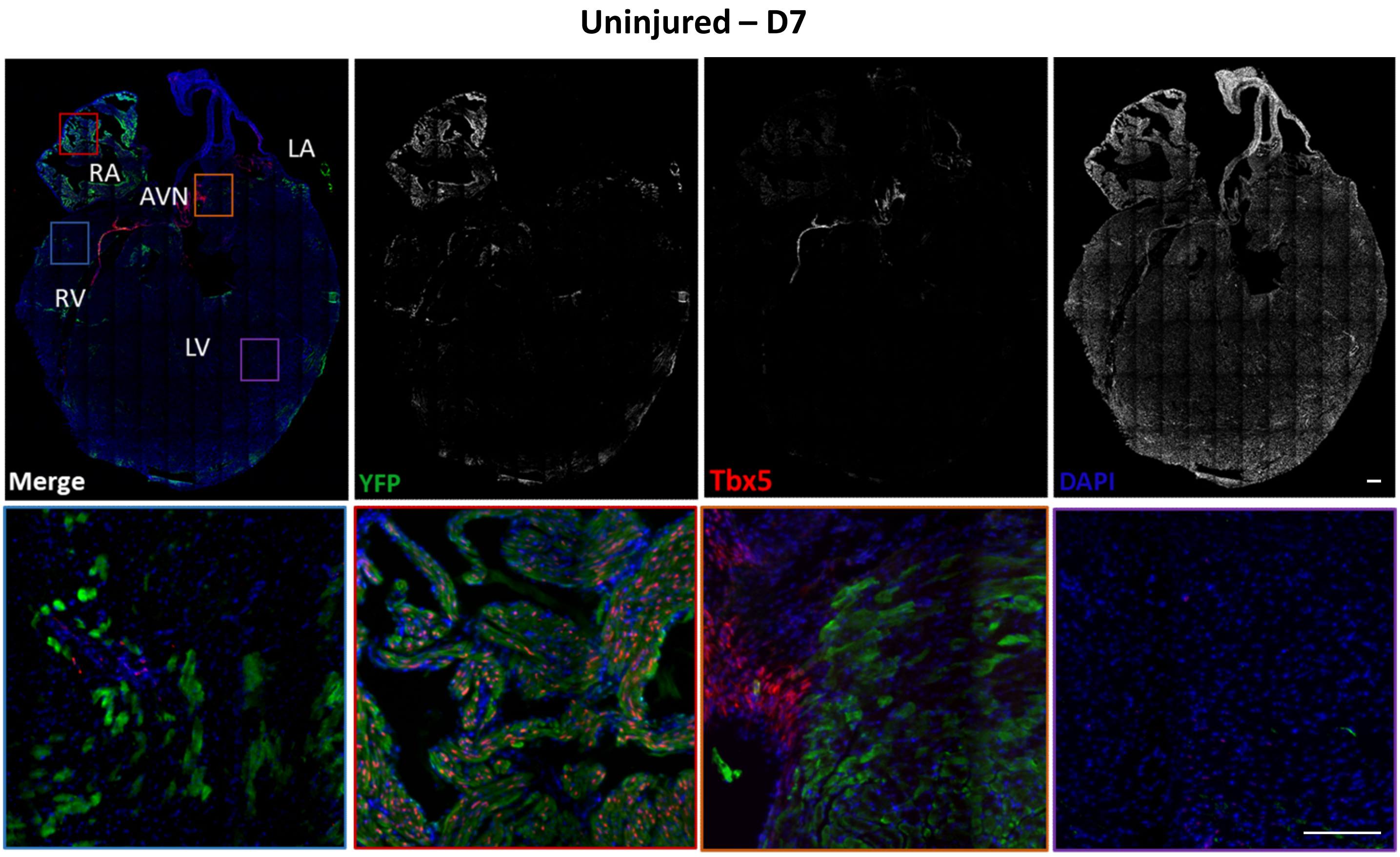

Supplementary Figure 3. A collage of an uninjured adult heart 7 days after receiving TAM. Inserts indicating (α-GFP) YFP^{+} and Tbx5^{+} are located in the Atria and AVN only. Key- LV=Left Ventricle, RV=Right Ventricle, RA=Right Atrium, LA=Left Atrium, AVN=Atrioventricular Node, N=2-3 hearts. Scale bar=100μm.

Supplementary Figure 4 **Siatra *et al.***

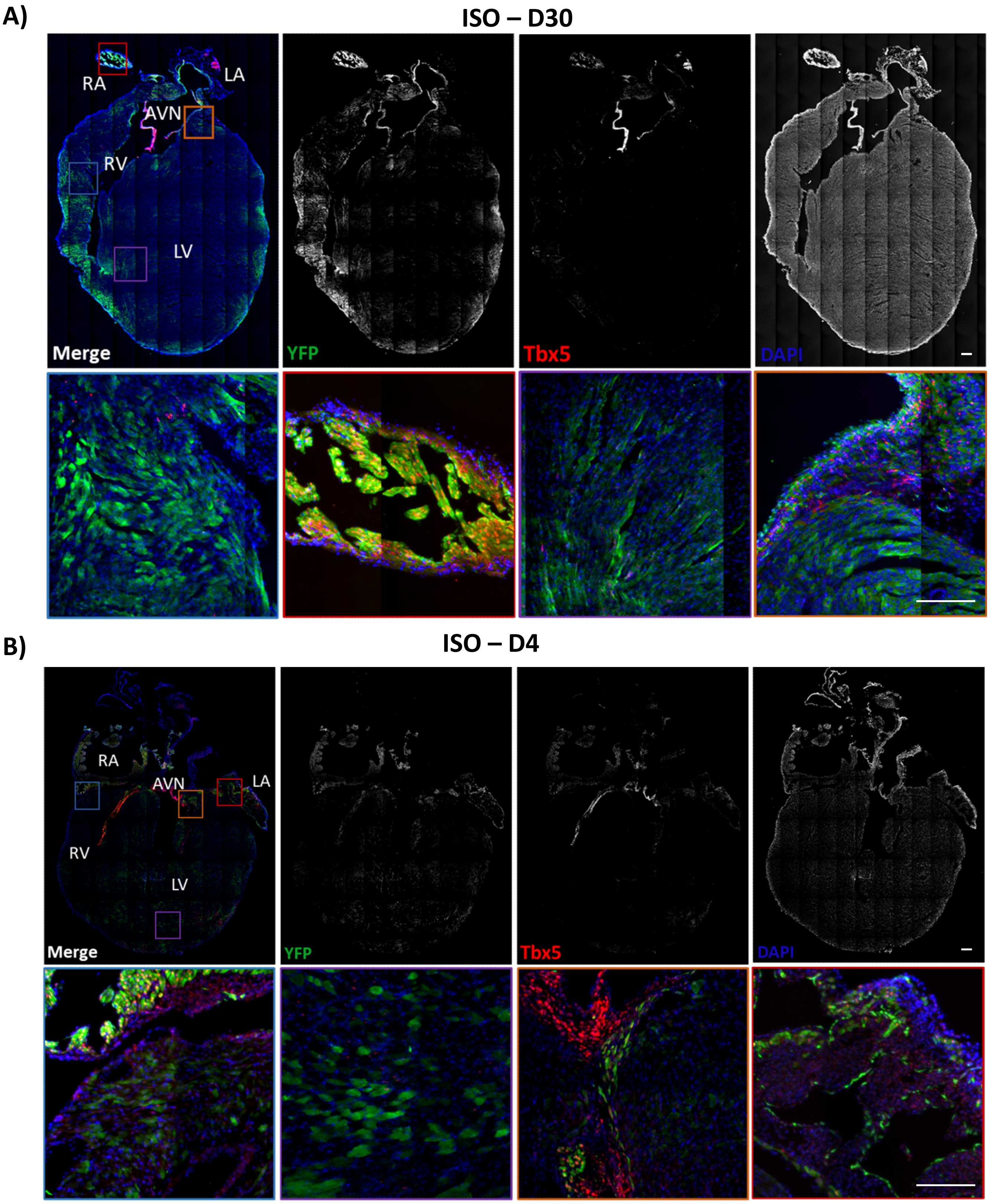

Supplementary Figure 4. (**A**) A collage of an ISO-injured adult heart on D30 after injury. (**B**) A collage of an ISO-injured adult heart on D4 after injury. Inserts indicating (α-GFP) $YFP^{+}Tbx5^{+}$ are located in the atria on both D4 and D30, while (α-GFP) $YFP^{+}Tbx5^{+}$ can be observed in D4 but not in D30 LV. N=1-3 hearts per timepoint. Key- LV=Left Ventricle, RV=Right Ventricle, RA=Right Atrium, LA=Left Atrium, AVN=Atrioventricular Node, SAN=Sinoatrial Node. Scale bar=100μm.

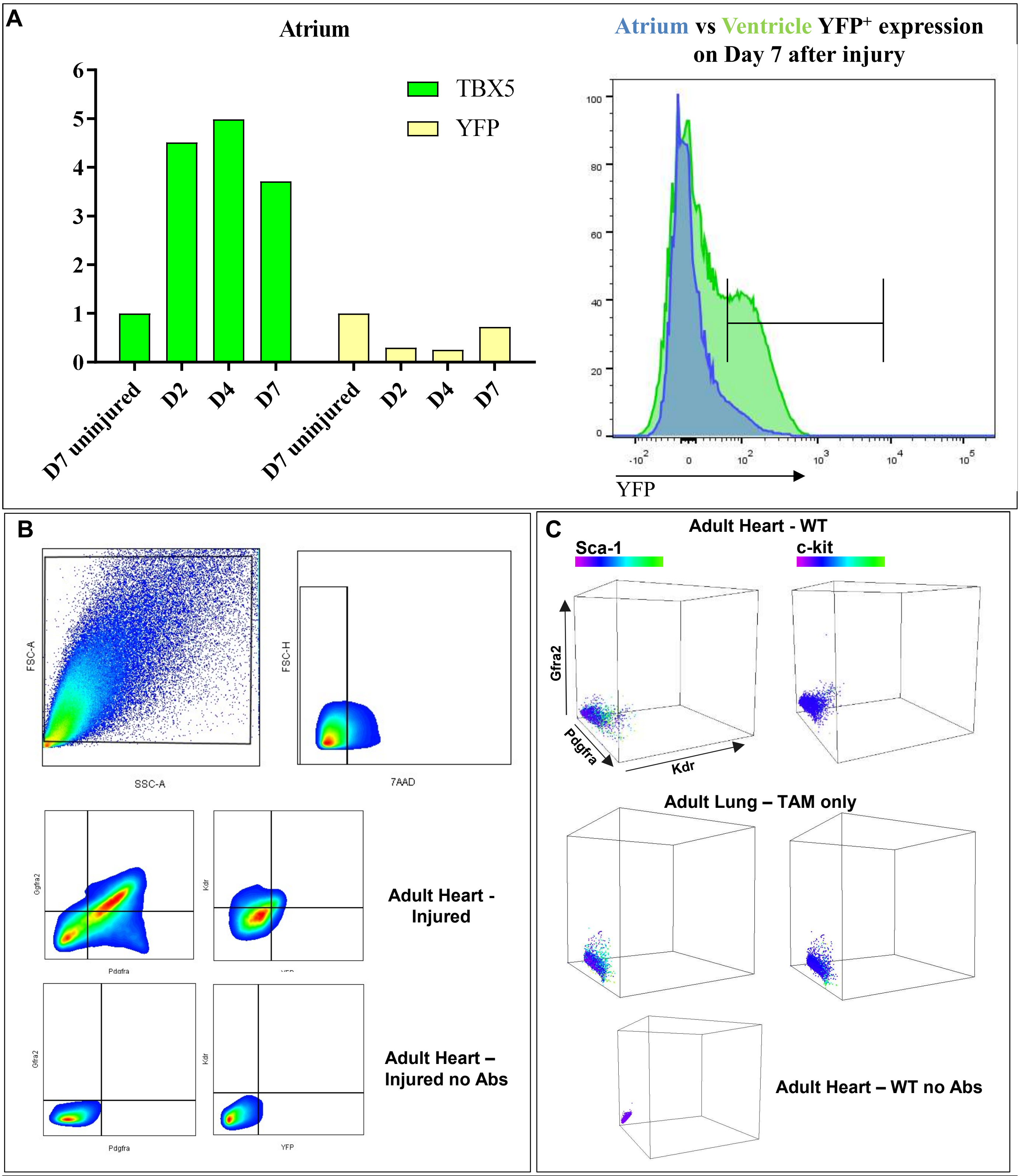

Supplementary Figure 5. (**A**) Real-time PCR analysis of *Yfp* and *Tbx5* transcripts in the atria of the adult heart in different time-points. Flow cytometry acquisition of YFP+ cells from atria and ventricles seven days post-injury. (**B**) Representative graphs of flow cytometric gating strategy analysis from cardiac cells collected from adult injured hearts. (**C**) Representative graphs of flow cytometry acquisition of cardiac cells from uninjured heart and lung tissue.

Supplementary Figure 6 – Tbx5 in heart homeostasis and pathophysiology **Siatra *et al.***

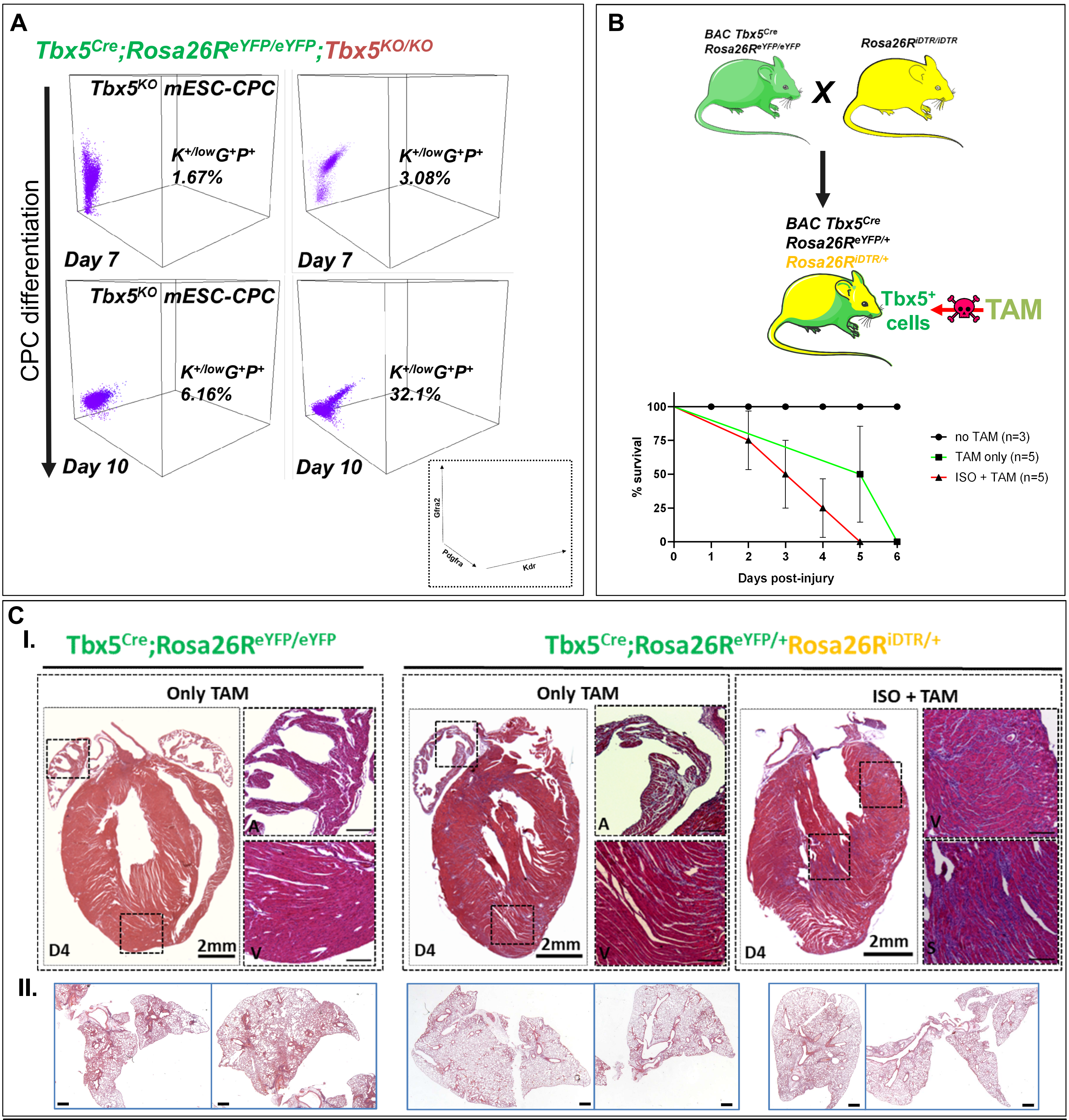

Supplementary Figure 6. (**A**) Representative three-dimensional graphs of flow cytometric acquisition of *Tbx5^{Cre}Rosa26R$^{eYFP/+}$Tbx5$^{KO/KO}$*-mESC CPC after 7 and 10 days under *in vitro* differentiating conditions. Percentages are depicted as triple-positive CPC in total live cells. (**B**) *Tbx5^{Cre}Rosa26R$^{eYFP/+}$Rosa26R$^{iDTR/+}$* cross and survival. Tamoxifen administration induces cells death to Tbx5^{+}-expressing cells. (**C**) (I.) Photos of adult hearts with haematoxylin and eosin staining confirmed non-fatal, yet substantial, heart injury in the left ventricle and atria, when compared to injured adult hearts. N=3 hearts per condition (II.) Photos of adult lung tissue with haematoxylin and eosin staining indicative of alveolar damage in DTA mice. N=1-2 lungs per condition. Scale bar=2mm. Error bars = SEM.

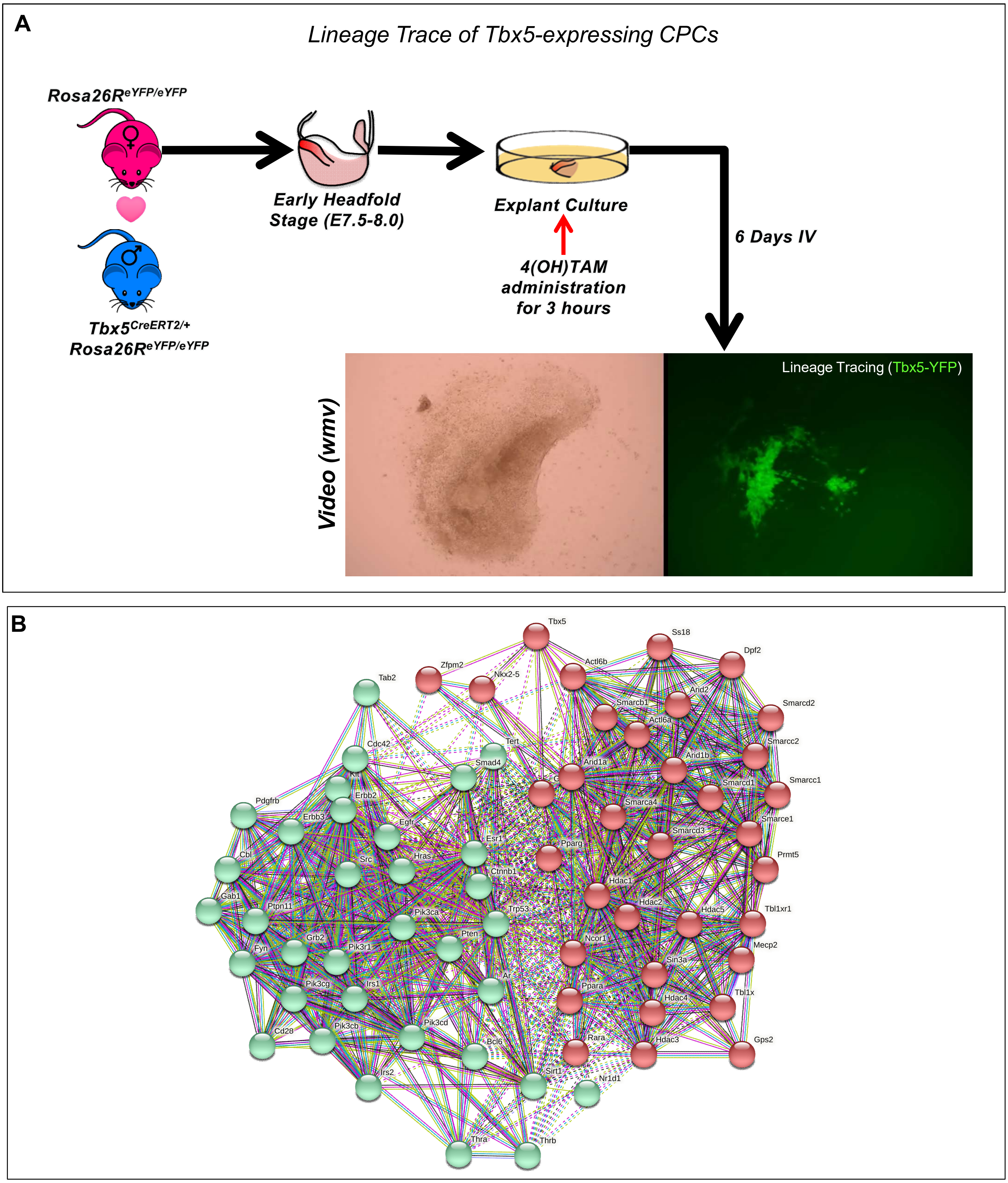

Supplementary Figure 7. (**A**) Non-beating cardiac tissue was extracted from EHF stage embryos, show beating *ex vivo* in the areas where Tbx5 (YFP) was expressed. Video indicates the beating of YFP⁺ cardiac cells. (**B**) *Kmeans* STRING transcriptional network diagram of *Mus musculus* Tbx5-related and Thrα/β proteins interactions.

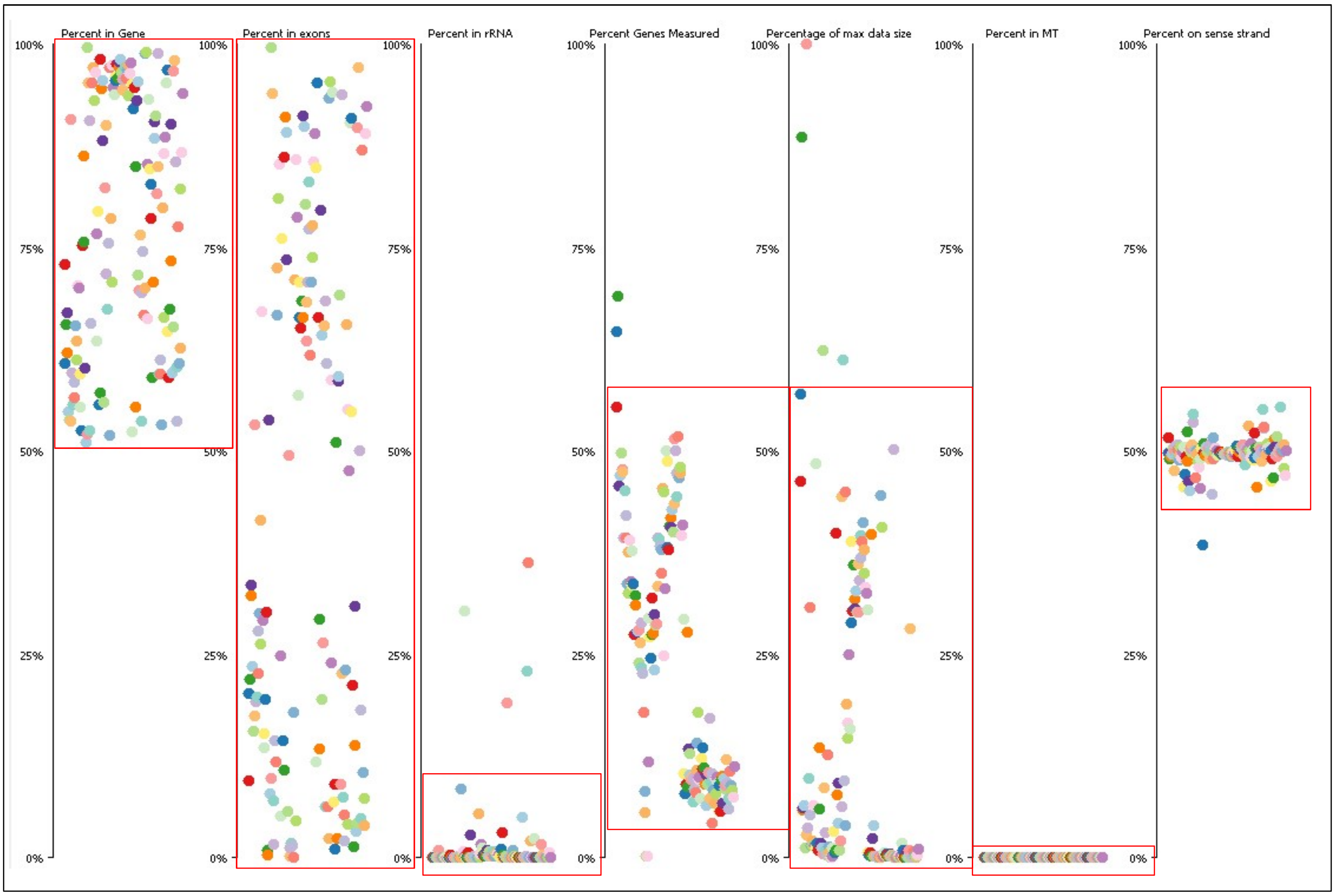

Supplementary Figure 8. Representative single-Cell RNA-seq QC report. In red, cut-off exclusion points for single-cells RNA-seq that have been chosen for downstream analysis (T-SNE, heatmaps, DEGs and GO/KEGG.

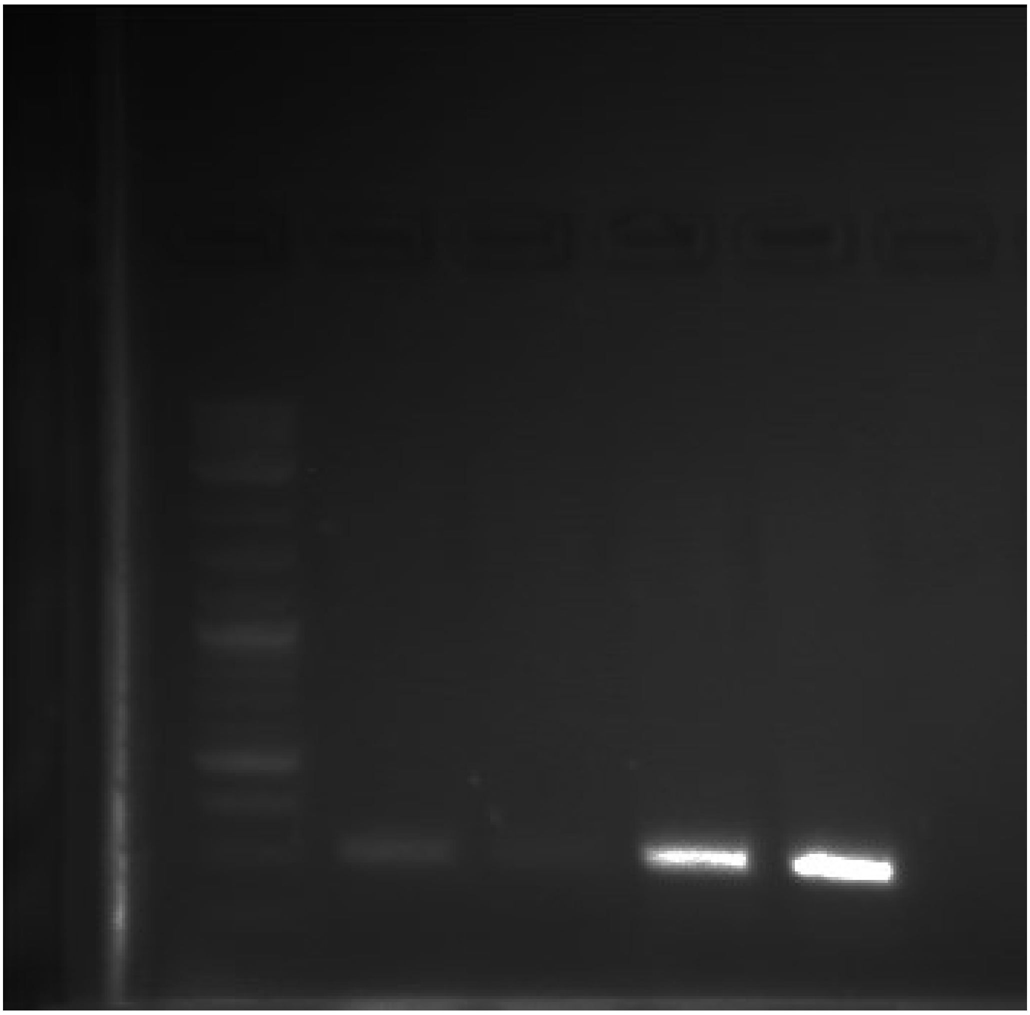

Supplementary Figure 9. Real-time PCR analysis of *Yfp* and *Tbx5* transcripts in the ventricles of the adult heart.

Supplementary Table 1 Siatra *et al.*

Input Parameter	Value
Single-end or paired-end reads	paired
Custom or built-in reference genome	indexed
Reference genome with or without an annotation	without-gtf
Select reference genome	mm10full
Gene model (gff3,gtf) file for splice junctions	
Length of the genomic sequence around annotated junctions	100
Use 2-pass mapping for more sensitive novel splice junction discovery	None
twopass_read_subset	Empty.
sj_precalculated	Empty.
Per gene/transcript output	-
Report chimeric alignments?	Don't report chimeric alignments
oformat	
Read alignment tags to include in the BAM output	NH (number of reported alignments/hits for the read) HI (query hit index) AS (local alignment score) nM (number of mismatches per (paired) alignment) ch (used to indicate chimeric alignments)
HI tag values should be	one-based
outSAMprimaryFlag	OneBestScore
MAPQ value for unique mappers	60
filter	
Exclude the following records from the BAM output	Nothing selected.
Would you like to set additional output filters?	yes
Would you like to keep only reads that contain junctions that passed filtering?	FALSE
Score range below the maximum score for multimapping alignments	1
Maximum number of alignments to output a read's alignment results, plus 1	10
Maximum number of mismatches to output an alignment, plus 1	10
Maximum ratio of mismatches to mapped length	0.3
Maximum ratio of mismatches to read length	1
Minimum alignment score	0
Minimum alignment score, normalized to read length	0
Minimum number of matched bases	20
Minimum number of matched bases, normalized to read length	0
Maximum number of multimapping alignments to output for a read	-1
Calculation method for TLEN	leftmost base of the (+)strand mate to rightmost base of the (-)mate. (+)sign for the (+)strand mate
Configure seed, alignment and limits options	full
seed	
Search start point through the read	30
Search start point through the read, normalized to read length	1
Maximum length of seeds	0
Maximum number of mappings to use a piece in stitching	10000
Maximum number of seeds per read	1000
Maximum number of seeds per window	50
Maximum number of one seed loci per window	10
align	
Minimum intron size	21
Maximum intron size	0
Maximum gap between two mates	0
Minimum overhang for spliced alignments	5
Minimum overhang for annotated spliced alignments	3
Minimum mapped length for a read mate that is spliced	0
Minimum mapped length for a read mate that is spliced, normalized to mate length	0.66
Maximum number of windows per read	10000
Maximum number of transcripts per window	100
Maximum number of different alignments per read to consider	10000
Use end-to-end read alignments, with no soft-clipping?	FALSE
minimum number of overlap bases to trigger mates merging and realignment	0
maximum proportion of mismatched bases in the overlap area	0.01
chim_settings	
Minimum length of chimeric segment	12
Minimum total (summed) score of chimeric segments	0
Maximum difference of chimeric score from read length	20
Minimum difference between the best chimeric score and the next one	10
Penalty for a non-GT/AG chimeric junction	-1
Minimum overhang for a chimeric junction	20
Maximum gap in the read sequence between chimeric segments	0
Discard chimeric alignments with Ns in the genome sequence around the chimeric junction	TRUE
Maximum number of multi-alignments for the main chimeric segment.	10
Maximum number of chimeric multi-alignments	1
Score range for multi-mapping chimeras	1
limits	
Maximum number of junctions for one read (including all multimappers)	1000
Maximum number of collapsed junctions	1000000
Maximum number of inserts to be inserted into the genome on the fly.	1000000
Number of genome bins for coordinate-sorting	50

Supplementary Table 1. Parameters for aligning FASTQ raw data on the mm10 mouse genome using the RNA-STAR tool in http://www.usegalaxy.eu
