# Supplementary material for: Return of the Tbx5; lineage-tracing reveals ventricular cardiomyocyte-like precursors in the injured adult mammalian heart
Source: NPJ Regen Med. 2023 Mar 3;8:13. doi: 10.1038/s41536-023-00280-9 (PMC9984483; doi:10.1038/s41536-023-00280-9)
Supplement: Supplementary file 3 — Reporting Summary [file 41536_2023_280_MOESM3_ESM.pdf]

nature portfolio

Corresponding author(s): Ioannis Kokkinopoulos

Last updated by author(s): Aug 10, 2022

Reporting Summary

Nature Portfolio wishes to improve the reproducibility of the work that we publish. This form provides structure for consistency and transparency in reporting. For further information on Nature Portfolio policies, see our Editorial Policies and the Editorial Policy Checklist.

Statistics

For all statistical analyses, confirm that the following items are present in the figure legend, table legend, main text, or Methods section.

n/a	Confirmed	
☐	☒	The exact sample size (*n*) for each experimental group/condition, given as a discrete number and unit of measurement
☐	☒	A statement on whether measurements were taken from distinct samples or whether the same sample was measured repeatedly
☐	☒	The statistical test(s) used AND whether they are one- or two-sided<br>*Only common tests should be described solely by name; describe more complex techniques in the Methods section.*
☒	☐	A description of all covariates tested
☐	☒	A description of any assumptions or corrections, such as tests of normality and adjustment for multiple comparisons
☐	☒	A full description of the statistical parameters including central tendency (e.g. means) or other basic estimates (e.g. regression coefficient) AND variation (e.g. standard deviation) or associated estimates of uncertainty (e.g. confidence intervals)
☐	☒	For null hypothesis testing, the test statistic (e.g. *F*, *t*, *r*) with confidence intervals, effect sizes, degrees of freedom and *P* value noted<br>*Give P values as exact values whenever suitable.*
☒	☐	For Bayesian analysis, information on the choice of priors and Markov chain Monte Carlo settings
☒	☐	For hierarchical and complex designs, identification of the appropriate level for tests and full reporting of outcomes
☐	☒	Estimates of effect sizes (e.g. Cohen's *d*, Pearson's *r*), indicating how they were calculated

Our web collection on statistics for biologists contains articles on many of the points above.

Software and code

Policy information about availability of computer code

Data collection	C1 fluidigm platform
Data analysis	www.useGalaxy.eu, SeqMonk 1.48.0, Partek FlowTM

For manuscripts utilizing custom algorithms or software that are central to the research but not yet described in published literature, software must be made available to editors and reviewers. We strongly encourage code deposition in a community repository (e.g. GitHub). See the Nature Portfolio guidelines for submitting code & software for further information.

Data

Policy information about availability of data

All manuscripts must include a data availability statement. This statement should provide the following information, where applicable:

- Accession codes, unique identifiers, or web links for publicly available datasets
- A description of any restrictions on data availability
- For clinical datasets or third party data, please ensure that the statement adheres to our policy

GSE63796

Human research participants

Policy information about studies involving human research participants and Sex and Gender in Research.

Reporting on sex and gender	N/A
Population characteristics	N/A
Recruitment	N/A
Ethics oversight	N/A

Note that full information on the approval of the study protocol must also be provided in the manuscript.

Field-specific reporting

Please select the one below that is the best fit for your research. If you are not sure, read the appropriate sections before making your selection.

☒ Life sciences ☐ Behavioural & social sciences ☐ Ecological, evolutionary & environmental sciences

For a reference copy of the document with all sections, see nature.com/documents/nr-reporting-summary-flat.pdf

Life sciences study design

All studies must disclose on these points even when the disclosure is negative.

Sample size	We initially used the G*power method to account for the animals used in this study. Yet, due to standardization, we exceeded that number
Data exclusions	We excluded some in vitro data due to poor culture outcome, as well as one of the reviewer's suggested experiment
Replication	All experiments have been reproduced and the variation has been noted, where appropriate.
Randomization	no randomization took place
Blinding	blinding was evident in both flow cytometry and in vivo data

Reporting for specific materials, systems and methods

We require information from authors about some types of materials, experimental systems and methods used in many studies. Here, indicate whether each material, system or method listed is relevant to your study. If you are not sure if a list item applies to your research, read the appropriate section before selecting a response.

Materials & experimental systems

n/a	Involved in the study	
☐	☒	Antibodies
☐	☒	Eukaryotic cell lines
☒	☐	Palaeontology and archaeology
☐	☒	Animals and other organisms
☒	☐	Clinical data
☒	☐	Dual use research of concern

Methods

n/a	Involved in the study	
☒	☐	ChIP-seq
☐	☒	Flow cytometry
☒	☐	MRI-based neuroimaging

Antibodies

Antibodies used	anti-human/mouse GFRA2 Polyclonal Goat IgG (R&D Systems), rat monoclonal anti-PDGFR beta antibody conjugated with PE (Abcam, APB5), donkey polyclonal anti-goat IgG Alexa 405 conjugated with UV (Abcam), rat monoclonal IgG2b anti-mouse KDR-Alexa647 conjugated with APC (BioLegend). 7-AAD (BioLegend, Cat no. 420404) was used as a viability marker. Sca-1 (Biolegend Cat no. 108127), c-Kit (Biolegend, Cat no. 105813), CD31 (Biolegend, Cat no. 102524), Cardiac troponin T (cTnT, 1/100, mouse monoclonal, Abcam), alpha smooth muscle actin (aSMA) (1/100, rabbit polyclonal, Abcam) and CD31 (1/25, Rabbit polyclonal, Abcam). For enhancing the endogenous YFP signal, in ICC, we used anti-GFP FITC-conjugated (1/100, goat polyclonal, Abcam). MF20 (mouse monoclonal, 1/100, Developmental Biology Hybridoma Bank), Tbx5 (rabbit polyclonal, 1/100, Sigma), GFRA2 (chicken polyclonal, 1/500, Antibodies-online, ABIN1450225), Connexin 43 (cat no C6219-.2ML, rabbit polyclonal, 1/2000, Sigma), α-actinin (A7811, clone

	EA-53, mouse monoclonal, 1/500, Sigma), Ki67 (ab15580, rabbit polyclonal, 1:100, Abcam). For enhancing the endogenous YFP signal in IHC, we used anti-GFP (chicken polyclonal, 1/1000, Abcam).
Validation	All antibodies applied, have been validated in relation to the species and technique used.

Eukaryotic cell lines

Policy information about cell lines and Sex and Gender in Research

Cell line source(s)	We used our in-house murine primary ESCs, described in Kokkinopoulos et al. 2016, PLoS ONE.
Authentication	This step occurred in Kokkinopoulos et al. 2016, PLoS ONE.
Mycoplasma contamination	Mycoplasma testing was performed once, indicating no mycoplasma contamination
Commonly misidentified lines (See ICLAC register)	N/A

Animals and other research organisms

Policy information about studies involving animals; ARRIVE guidelines recommended for reporting animal research, and Sex and Gender in Research

Laboratory animals	Adult (both female and male) two-three month-old Tbx5CreERT2/Rosa26ReYFP/eYFP, Tbx5CreERT2/+/Rosa26ReYFP/+/Rosa26RiDTR/+ were employed. These animals were bred on a mixed background.
Wild animals	N/A
Reporting on sex	N/A
Field-collected samples	N/A
Ethics oversight	All animal work has been approved by the BRFAA ethics committee and the Attica Veterinary Department (Animal Licence; 60876/23-1-20)

Note that full information on the approval of the study protocol must also be provided in the manuscript.

Flow Cytometry

Plots

Confirm that:

☒ The axis labels state the marker and fluorochrome used (e.g. CD4-FITC).

☒ The axis scales are clearly visible. Include numbers along axes only for bottom left plot of group (a 'group' is an analysis of identical markers).

☒ All plots are contour plots with outliers or pseudocolor plots.

☒ A numerical value for number of cells or percentage (with statistics) is provided.

Methodology

Sample preparation	Cultured cells were treated with 0.05% trypsin/EDTA (Gibco) for 5 min at 37°C under 5% CO2. Prior to Ab staining, cells were blocked with 5% FBS in 1X PBS for 20'.
Instrument	ARIA II Analyzer (BD Biosciences)
Software	FACSDiva 7.0 software
Cell population abundance	7-AAD (BioLegend, Cat no. 420404) was used as a viability marker
Gating strategy	gating strategy is described in the supplementary methods

☒ Tick this box to confirm that a figure exemplifying the gating strategy is provided in the Supplementary Information.
